# Time-resolved luminescence detection of peroxynitrite using a reactivity-based lanthanide probe†

**DOI:** 10.1039/c9sc06053g

**Published:** 2020-02-18

**Authors:** Colum Breen, Robert Pal, Mark R. J. Elsegood, Simon J. Teat, Felipe Iza, Kristian Wende, Benjamin R. Buckley, Stephen J. Butler

**Affiliations:** Department of Chemistry, Loughborough University Epinal Way Loughborough LE11 3TU UK S.J.Butler@lboro.ac.uk; Department of Chemistry, Durham University South Road Durham DH1 3LE UK; Advanced Light Source, Berkeley Lab. 1 Cyclotron Road Berkeley CA 94720 USA; Centre for Biological Engineering, Department of Mechanical, Electrical and Manufacturing Engineering, Loughborough University LE11 3TU UK; Leibniz-Institute for Plasma Science and Technology, ZIK plasmatis Felix-Hausdorff-Str.2 17489 Greifswald Germany

## Abstract

Peroxynitrite (ONOO^−^) is a powerful and short-lived oxidant formed *in vivo*, which can react with most biomolecules directly. To fully understand the roles of ONOO^−^ in cell biology, improved methods for the selective detection and real-time analysis of ONOO^−^ are needed. We present a water-soluble, luminescent europium(iii) probe for the rapid and sensitive detection of peroxynitrite in human serum, living cells and biological matrices. We have utilised the long luminescence lifetime of the probe to measure ONOO^−^ in a time-resolved manner, effectively avoiding the influence of autofluorescence in biological samples. To demonstrate the utility of the Eu(iii) probe, we monitored the production of ONOO^−^ in different cell lines, following treatment with a cold atmospheric plasma device commonly used in the clinic for skin wound treatment.

## Abstract 2

Reactivity-based europium(iii) probe displays excellent selectivity for peroxynitrite (ONOO^−^), enabling its time-resolved luminescence detection in living cells.

## Graphical abstract

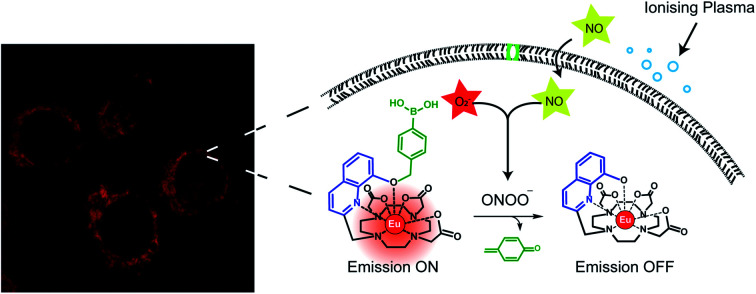

## Introduction

Peroxynitrite (ONOO^−^) is a highly reactive nitrogen species generated from the spontaneous reaction between superoxide (O_2_^−^) and nitric oxide (NO). In cells, ONOO^−^ is generated predominantly in the mitochondria and is involved in cellular signalling processes, *via* nitration of tyrosine residues on proteins and nitrosylation of protein thiols.^1–4^ In contrast, elevated levels of ONOO^−^ can lead to significant oxidative and nitrosative damage to lipids, proteins and DNA and has been implicated in several diseases including Alzheimer's, Huntington's, and Parkinson's disease.^5–7^ Therefore, ONOO^−^ has become a very important target for detection in a cellular environment.

The ability to detect ONOO^−^ selectively would facilitate a better understanding of its roles in cellular biology, disease diagnosis, and therapies based on redox signalling events (*e.g.* photodynamic therapy and radiotherapy).^8^ In recent years, cold atmospheric plasma (CAP) has emerged as a promising new biomedical technology for the treatment of chronic wounds and cancer (*e.g.* breast, skin, lung, and pancreatic cancer).^9–11^ The therapeutic effects of CAP are linked to the high concentrations of reactive oxygen and nitrogen species (RONS) generated by the plasma.^12^

In chronic wound treatment, plasma generated NO and ONOO^−^ are proposed to break down bacterial biofilm by interacting with the lipid membrane, forming pores that facilitate further RONS ingress.^13,14^ The selective killing of cancer cells by plasma has been attributed to high levels of RONS (*e.g.* NO, ONOO^−^, H_2_O_2_), which exceeds the cancer cell's compromised oxidative defence mechanisms.^9^ Despite the importance of RONS in plasma-based cell therapy, very little is known about the specific interactions between plasma and biological samples. To elucidate the mechanisms that deliver plasma generated RONS to cells and tissues, improved methods for the precise measurement of specific RONS are needed.

Detecting ONOO^−^ is particularly difficult due to its very short lifetime (10 ms) and propensity to react, forming longer-lived species (NO_3_^−^). A traditional assay for ONOO^−^ detection involves immunostaining of 3-nitrotyrosine;^15^ however, this approach is not applicable for live-cell imaging studies. In recent years, fluorescent molecular probes have emerged as attractive tools for imaging ONOO^−^ in a cellular environment. A series of reactivity-based probes have been devised that signal ONOO^−^ by a change in fluorescence.^16*a*–*e*^ Notwithstanding some notable exceptions,^16*b*,*e*^ the majority of existing ONOO^−^ probes have limitations in performance, due to their lack of solubility in 100% aqueous media, limited selectivity for ONOO^−^ over other RONS (*e.g.* NO, hypochlorite, hydroxyl radicals), and relatively slow response times, which prevents real-time measurements. One major difficulty encountered with organic probes stems from their short-lived fluorescence, which can be difficult to distinguish from the background fluorescence of biomolecules, often precluding accurate measurements of ONOO^−^.^17^

Luminescent lanthanide probes offer unique photophysical advantages over classical organic fluorescent probes,^18,19^ including: (1) long emission lifetimes that enable time-gated and time-resolved measurements, thereby increasing precision and signal to noise;^20,21^ (2) well-defined emission bands that may vary in intensity in response to specific analytes, allowing quantitative analysis;^22^ and (3) large separations between the absorption and emission spectra, which minimises self-quenching processes. Several water-soluble lanthanide complexes have been developed into effective cellular imaging probes, which localise to specific subcellular compartments and report on target species with high spatial and temporal resolution.^23,24^ A few lanthanide probes have been developed to detect ONOO^−^;^25,26^ however, their sensing mechanisms rely upon quenching of an excited state complex, involving charge transfer with the electron rich ONOO^−^. Such probes are limited in cellular imaging applications because other electron rich species present in the cell (*e.g.* urate, ascorbate) are known to induce excited state quenching,^27,28^ and could also give rise to the observed decrease in luminescence.

**Fig. 1:**
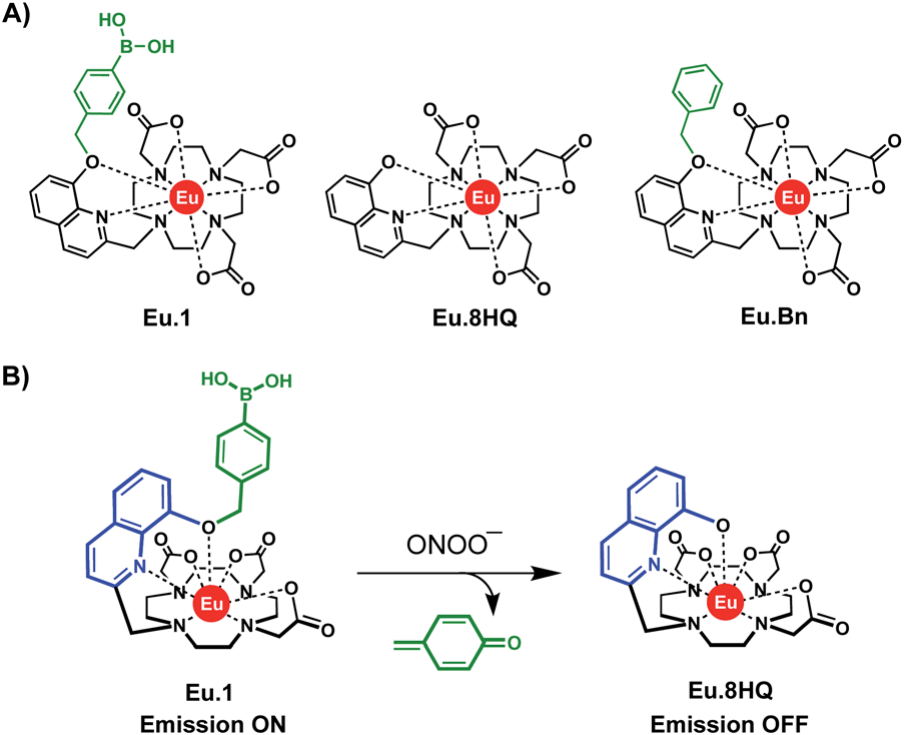
(A) Structure of Eu(iii) probe **Eu.1** and control complex **Eu.Bn**. (B) Mechanism of detection of ONOO^−^, based on oxidative cleavage of the phenyl boronic acid group of **Eu.1** to form non-emissive complex **Eu.8HQ**.

In this work, we report the design and synthesis of a water-soluble, luminescent Eu(iii) complex, **Eu.1** (Fig. 1A) for the time-resolved detection of ONOO^−^ in human blood serum and living cells. The probe comprises a quinoline antenna functionalised at the 8-position with a benzyl boronic acid, which undergoes rapid oxidative cleavage in the presence of ONOO^−^ to reveal an 8-hydroxyquinoline unit, which shuts down energy transfer to the Eu(iii) ion and switches off luminescence (Fig. 1B). **Eu.1** displays nanomolar sensitivity to ONOO^−^ and provides a long-lived luminescence signal, which permits time-resolved measurements of ONOO^−^, completely eliminating background autofluorescence from the biological sample. To demonstrate its utility in cellular applications, the probe was used to quantify ONOO^−^ in different cell lines, following treatment with a commercial plasma device, CAP jet kINPEN® MED, clinically used to foster chronic and acute wound healing.

## Results and discussion

### Probe design, synthesis and characterisation

Probe **Eu.1** is based on a nonadentate macrocyclic ligand, possessing a single coordinating quinoline group that acts as an efficient sensitizer of Eu(iii) emission. The quinoline unit binds to the Eu(iii) ion in a bidentate manner, effectively shielding the metal centre from surrounding water molecules, thereby enhancing the emission intensity and lifetime of **Eu.1**. The probe is functionalised with a benzyl boronic acid group, which we envisaged would react rapidly and selectively with ONOO^−^, causing oxidative cleavage to reveal an 8-hydroxyquinoline unit, which prevents energy transfer to the Eu(iii) ion and triggers a decrease in time-resolved luminescence (Fig. 1B). The probe exploits the considerably higher reactivity of phenyl boronic acids towards ONOO^−^ (*k* ≈ 10^6^ M^−1^ s^−1^) at physiological pH, compared with other RONS (*e.g.* H_2_O_2_, *k* ≈ 2.2 M^−1^ s^−1^).^29^ A control complex, **Eu.Bn** (Fig. 1A), lacking the boronic acid moiety, was synthesised to validate the response of **Eu.1** towards ONOO^−^. Additionally, **Eu.8HQ**, the expected product of the reaction between **Eu.1** and ONOO^−^, was synthesised to confirm its lack of luminescence in water.

Full details of the synthesis of **Eu.1**, **Eu.Bn** and **Eu.8HQ** are provided in the ESI.† Briefly, **Eu.1** was synthesised starting from 8-hydroxyquinoline-2-carboxaldehyde, which was *O*-alkylated with 4-iodobenzyl bromide, followed by reduction of the aldehyde to the primary alcohol. Subsequent mesylation of the methyl alcohol and alkylation onto a *tert*-butyl protected DO3A gave the macrocyclic ligand, which was deprotected and complexed with EuCl_3_. The Eu(iii) complex was subjected to a Miyaura borylation to provide **Eu.1** after purification by reverse-phase HPLC.

**Fig. 2:**
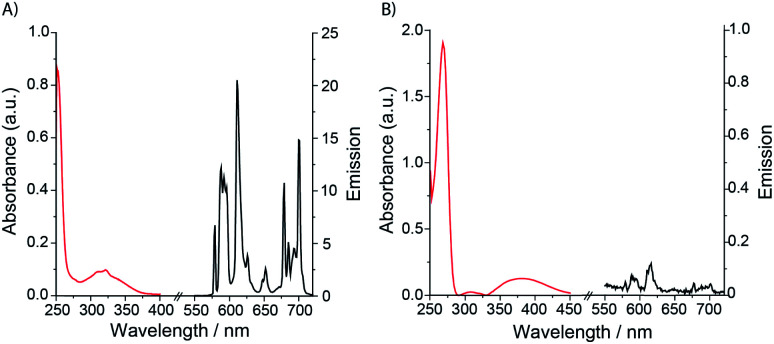
Absorption and emission spectra of (A) **Eu.1** (25 μM, *λ*_exc_ = 321 nm, *λ*_em_ = 550–720 nm); (B) **Eu.8HQ** (25 μM, *λ*_exc_ = 384 nm, *λ*_em_ = 550–720 nm), measured in aqueous buffer (100 mM PBS, pH 7.4).

Selected photophysical data for **Eu.1**, **Eu.Bn**, and **Eu.8HQ** are provided in Table 1. The UV-Vis absorption spectra of **Eu.1** and **Eu.Bn** in water are similar and feature a broad band centred at 321 nm (Fig. 2A). In contrast, **Eu.8HQ** displays a significantly red-shifted band centred at 384 nm (Fig. 2B), which may be associated with a charge-transfer transition, involving transfer of charge density from the quinoline oxygen atom to the quinoline unit.^32^ The emission properties of **Eu.1** and **Eu.8HQ** in buffered aqueous solution (100 mM PBS, pH 7.4) were very different (Fig. 2). **Eu.1** displayed significant Eu(iii) centred luminescence with a quantum yield of 10% (*λ*_exc_ = 321 nm). The emission spectrum is characterised by three components in the Δ*J* = 1 band (585–605 nm), indicating a structure of low symmetry, and a prominent Δ*J* = 2 (605–630 nm) band. In contrast, **Eu.8HQ** was found to be very weakly emissive (*ϕ*_em_ < 0.5%) over the pH range 4–10 (Fig. 2B and S2†), when excited at either 321 nm or 384 nm. The weak luminescence of **Eu.8HQ** can be attributed to the low lying excited state of the 8-hydroxyquinolinate group (around 17 100 cm^−1^),^33^ and its inability to effectively populate the Eu(iii) excited state.^34^ The free ligand **8HQ** is also nonfluorescent (Fig. S3†), consistent with efficient non-radiative decay of the 8-hydroxyquinoline excited state, due to intermolecular proton transfer.^35,36^ In support of this hypothesis, strong fluorescence was observed for the iodo-ligand precursor to **Eu.1** (Fig. S3†), in which the benzyl ether prevents non-radiative energy loss *via* a proton transfer mechanism.

**Table 1:** Photophysical data for Eu(iii) complexes (100 mM PBS, pH 7.4)

Complex	*λ*_max_/nm	*ε*/M^−1^ cm^−1^	*ϕ*_em_ a/%	*τ*_H_2_O_/ms	*τ*_D_2_O_/ms	*q* b
**Eu.1**	321	2800	10	0.52	0.66	0.2
**Eu.8HQ**	382	2100	<0.5	0.33	0.42	0.5
**Eu.Bn**	321	2600 (2560)	6 (6)	0.58 (0.37)	0.72 (0.44)	0.1 (0.2)

a Errors in quantum yields and lifetimes are ±15%.

b Values of hydration state, *q* (±20%) were derived using literature methods.^30^ Values for **Eu.Bn** in parentheses were determined in water previously, by Williams.^31^

**Fig. 3:**
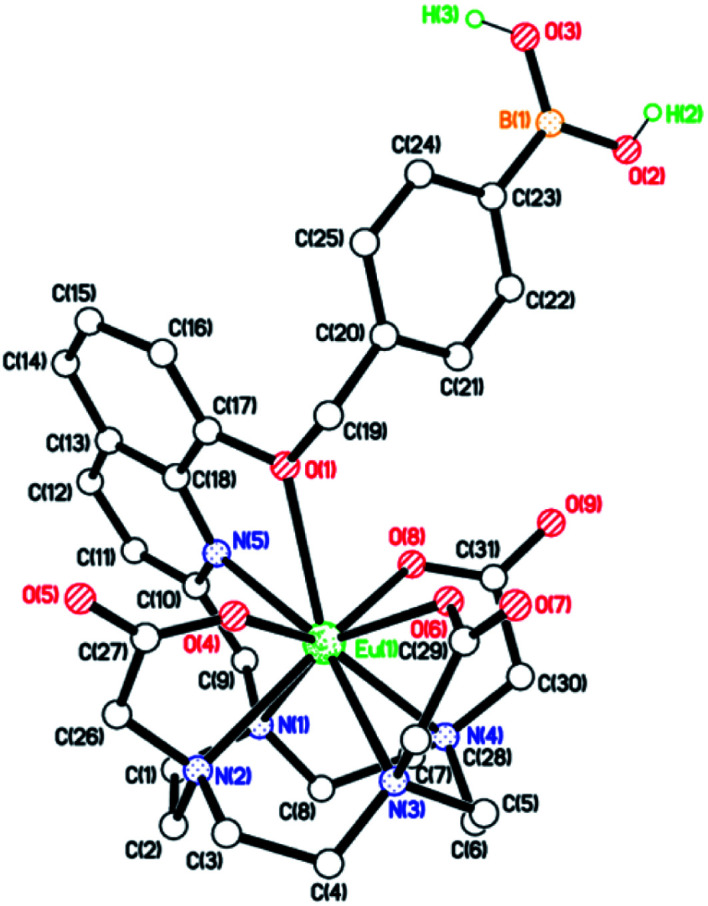
X-ray crystal structure of **Eu.1**, revealing a lack of water in the first coordination sphere of Eu(iii).

**Eu.1** possesses a long luminescence lifetime in water (0.52 ms), which increases slightly in D_2_O (0.66 ms). The hydration state (*q* value) of **Eu.1** was calculated to be 0.2,^30^ indicating a lack of coordinated water in the first coordination sphere. This was supported by synchrotron X-ray analysis of the crystal structure of **Eu.1** (Fig. 3; CCDC 1965719, see ESI† for further details), grown from slow evaporation of an aqueous solution. The Eu(iii) ion is encapsulated by the nonadentate ligand in a twisted square antiprismatic geometry, coordinated to four nitrogen atoms from the macrocyclic ring, three oxygen atoms from the carboxylate groups, one nitrogen from the quinoline group and one axial oxygen atom from the quinoline 8-alkoxyl group, inhibiting water access. The nearest water molecules are over 6.5 Å away from the Eu(iii) ion (Fig. S4†); such effective shielding from water increases the luminescence of **Eu.1**, by minimising deactivation of the Eu(iii) excited state *via* energy transfer to O–H vibrations. The structurally related complex, **Eu.Bn**, has a hydration state close to zero, and is expected to adopt a similar coordination geometry to that of **Eu.1**, in which the benzyloxy group occupies the axial position, preventing water binding.^31^ For **Eu.8HQ**, a *q* value of 0.5 was found, indicating ‘partial hydration’ of the complex,^30^ in which a water molecule is bound in the second hydration sphere, possibly held by hydrogen bonds to the quinoline 8-hydroxyl group.

### Evaluation of **Eu.1** for selective detection of peroxynitrite

**Fig. 4:**
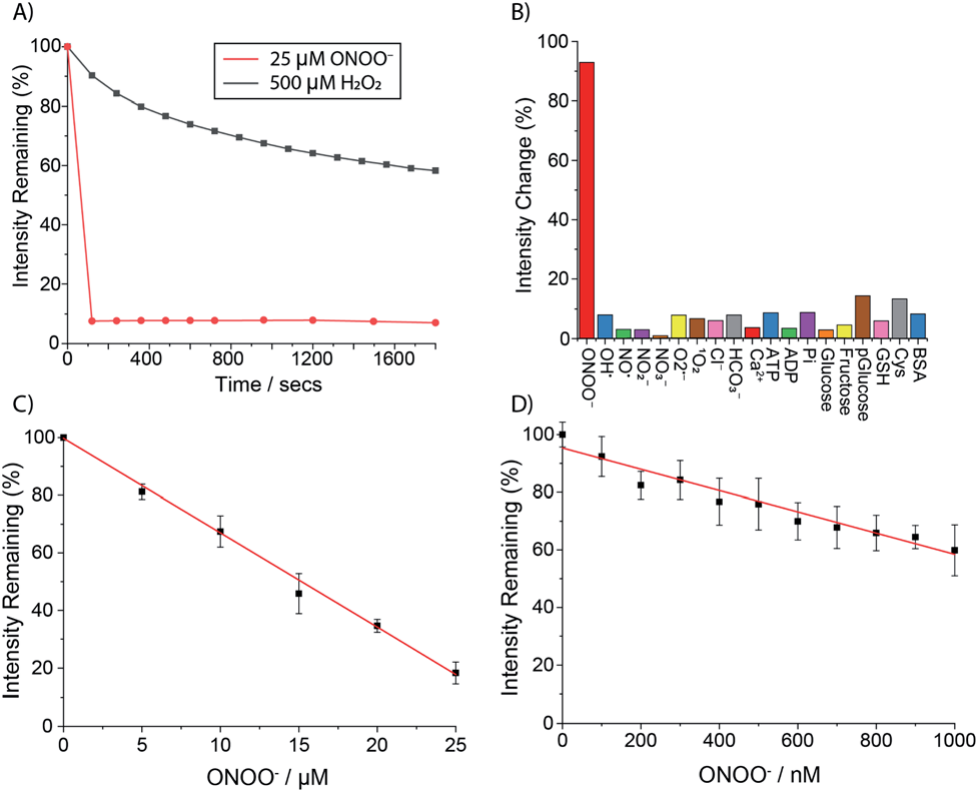
(A) Immediate and dramatic response of **Eu.1** to ONOO^−^ (25 μM, red) compared with the gradual response to H_2_O_2_ (500 μM, black), measured in 100 mM PBS, pH 7.4 (*λ*_ex_ = 321 nm); (B) selective response of **Eu.1** (25 μM) towards ONOO^−^ (25 μM), over other RONS including 100 μM HO˙, NO, NO_2_^−^, NO_3_^−^, O_2_˙^−^, ^1^O_2_, as well as biological analytes (1 mM Cl^−^, HCO_3_^−^, Ca^2+^, ATP, ADP, phosphate, glucose, fructose, phosphoglucose, glutathione, cysteine, and BSA), recorded after 30 minute incubation in 100 mM PBS, pH 7.4 (*λ*_ex_ = 321 nm, *λ*_em_ = 605–630 nm); (C) calibration plot for the time-resolved detection of micromolar (1–25 μM) ONOO^−^ using **Eu.1** (25 μM); and (D) calibration plot for the time-resolved detection of nanomolar (1–1000 nM) ONOO^−^ using **Eu.1** (5 μM) (*λ*_ex_ = 321, *λ*_em_ = 605–630 nm, integration time 60–400 μs).

The ability of **Eu.1** to detect peroxynitrite was evaluated in aqueous buffer (100 mM PBS pH 7.4). Addition of 1 equivalent of ONOO^−^ to **Eu.1** (25 μM) resulted in immediate and almost complete loss of Eu(iii) luminescence (Fig. 4A), consistent with rapid oxidative cleavage of the benzyl boronic acid group and quantitative formation of the weakly emissive complex **Eu.8HQ**. This was confirmed by UV-Vis analysis of the reaction mixture, which showed a characteristic red-shifted band centred at 384 nm, and LC-MS revealed a single peak with *m*/*z* = 654.1 corresponding to the single charged complex [**Eu.8HQ** + H]^+^ (Fig. S5†). The fast sensing kinetics of **Eu.1** is an important feature given the transient nature of ONOO^−^ in biological systems.

In contrast, treatment of **Eu.1** with 1 equivalent of H_2_O_2_ (25 μM), the longest-lived reactive species *in cellulo*,^37^ caused a minor (approximately 5%) decrease in emission intensity (Fig. S6†), and a much higher concentration of H_2_O_2_ (500 μM) was required to induce a gradual 45% decrease in emission intensity after 20 minutes (Fig. 4A). Treatment of the control complex **Eu.Bn** with ONOO^−^ or H_2_O_2_ under the same conditions resulted in less than 15% change in emission intensity and negligible changes in absorption spectra (Fig. S7†).

The high selectivity of **Eu.1** for ONOO^−^ was demonstrated fully by incubating the probe with a range of RONS (100 μM HO˙, NO, NO_2_^−^, NO_3_^−^, O_2_˙^−^, ^1^O_2_), as well as common biological cations (*e.g.* 1 mM Mg^2+^, Ca^2+^), anions (1 mM ATP, ADP bicarbonate, chloride) and biomolecules (*e.g.* BSA, glutathione) (Fig. 4B). The only species that induced a significant (>20%) change in luminescence of **Eu.1** was hypochlorite (OCl^−^); addition of 50 μM OCl^−^ caused a 50% decrease in emission intensity of **Eu.1** (Fig. S9†). Considering that the concentration of OCl^−^ in cells is estimated to be less than 1 μM,^38^ and that it reacts rapidly with endogenous thioethers and amines (*e.g.* Met = 3.8 × 10^7^, His = 1.0 × 10^5^ M^−1^ s^−1^),^39,40^ it can be assumed that OCl^−^ will not compete for **Eu.1** in a cellular environment.

The sensitivity of **Eu.1** towards ONOO^−^ was evaluated by incubating the probe with different concentrations of ONOO^−^ and recording the time-resolved emission intensity using a plate reader. Pleasingly, the linear decrease in time-resolved luminescence of **Eu.1** can be used to detect ONOO^−^ in the nanomolar concentration range (1–1000 nM) (Fig. 4D). The probe is thus well suited for measuring steady state concentrations of intracellular ONOO^−^, estimated to be in the nanomolar range.^41^ Further, the dynamic range of ONOO^−^ detection can be tuned by varying the concentration of **Eu.1**: incremental addition of ONOO^−^ (1–25 μM) to **Eu.1** (25 μM) provided a linear calibration plot (Fig. 4C), suitable for the measurement of micromolar levels of ONOO^−^. Such levels may be generated in experiments designed to administer reactive species to cells and tissues (*e.g.* by using ionizing plasma).^42–46^

### Time-resolved measurement of peroxynitrite in human serum

**Fig. 5:**
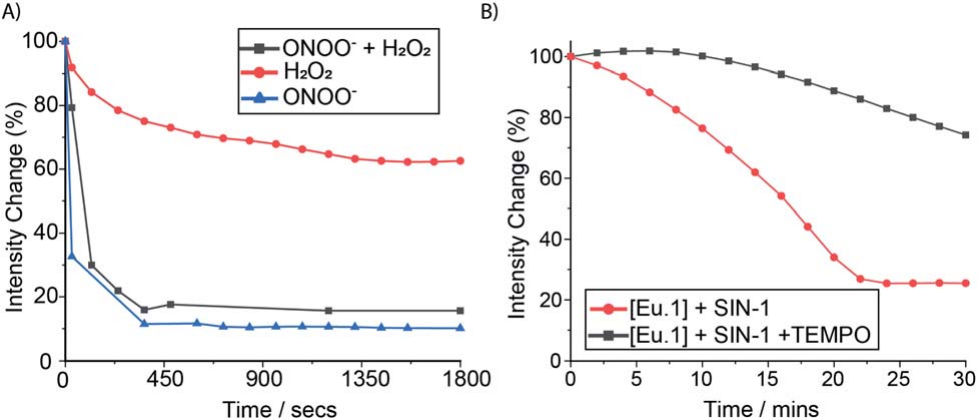
Time-resolved measurement of ONOO^−^ in human serum. (A) Change in emission intensity of **Eu.1** (50 μM) following incubation with H_2_O_2_ (500 μM, red), ONOO^−^ (50 μM, blue), and ONOO^−^ plus H_2_O_2_ (50 μM and 500 μM respectively, grey). (B) Time-dependent change in emission intensity of **Eu.1** (50 μM) following incubation with 500 μM SIN-1 (red), and SIN-1 plus 500 μM TEMPO (black), measured in human serum (pH 7.0–7.4), *λ*_ex_ = 321, *λ*_em_ = 605–630 nm, integration time 60–400 μs.

To further demonstrate the high sensitivity and selectivity of **Eu.1** for ONOO^−^, a competition experiment was undertaken in human serum, containing excess H_2_O_2_. The time-resolved emission response of **Eu.1** towards ONOO^−^, in the absence and presence of 500 μM H_2_O_2_, was essentially the same (Fig. 5A), revealing the ability of **Eu.1** to detect ONOO^−^ in biological media, in a background of excess H_2_O_2_. Next, the production of ONOO^−^ in human serum was monitored in real time, by incubating **Eu.1** with 3-morpholinosydnonimine (SIN-1, 250 μM), which decomposes to release equimolar amounts of NO and O_2_˙^−^, leading to spontaneous and sustained production of ONOO^−^ (mimicking cellular ONOO^−^ production). Incubation with SIN-1 caused a 75% reduction in time-resolved luminescence of **Eu.1** after approximately 20 minutes (Fig. 5B), corresponding to the formation of approximately 40 μM ONOO^−^, determined from the linear calibration plot (Fig. S8†). Further, the simultaneous addition of SIN-1 and the superoxide scavenger, TEMPO (2,2,6,6-tetramethylpiperidine-*N*-oxyl), resulted in a significantly smaller (25%) decrease in time-resolved emission of **Eu.1** over a 30 minute period (Fig. 5B), corresponding to the formation of 10 μM ONOO^−^ under these conditions.

### Cellular uptake and localisation studies

**Fig. 6:**
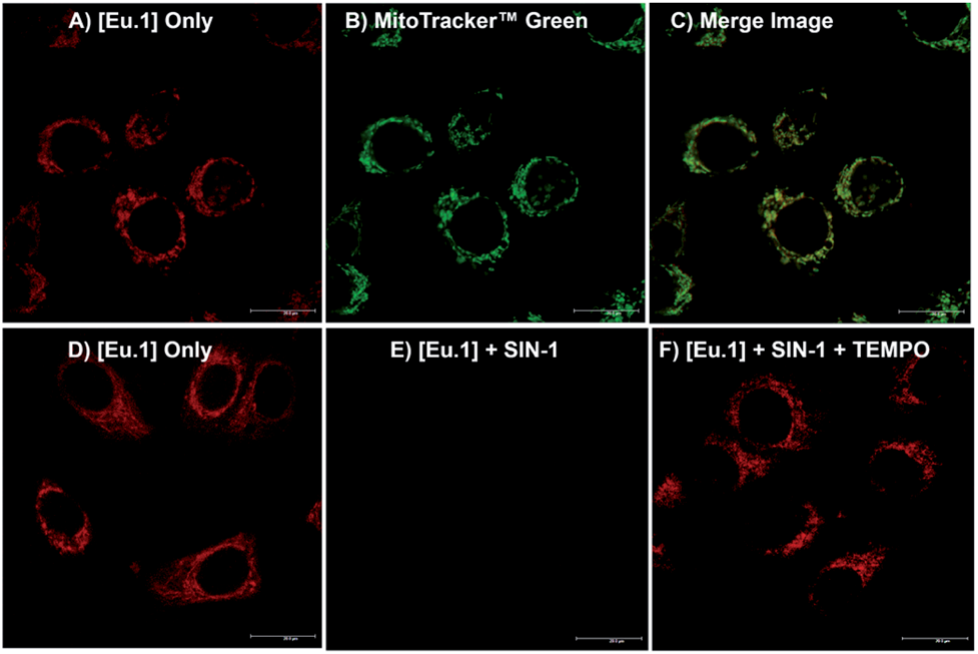
LSCM images showing localisation of **Eu.1** in the mitochondria of HeLa cells. (A) **Eu.1** only (250 μM, 4 h incubation); (B) MitoTracker Green™; and (C) merge image (*P* = 0.80). (D) LSCM image of **Eu.1** (250 μM, 4 h); (E) **Eu.1** (250 μM, 4 h) followed by SIN-1 (500 μM, 30 minute incubation); and (F) **Eu.1** (250 μM, 4 h) following incubation with SIN-1 and TEMPO (500 μM and 1 mM respectively, 30 minute incubation).

Having shown that **Eu.1** can be used to measure the production of micromolar levels of ONOO^−^ in human serum, by gating out short-lived autofluorescence from biological fluorophores, we next investigated the ability of **Eu.1** to measure dynamic changes in ONOO^−^ concentration in living cells. First, the cellular uptake behaviour of **Eu.1** in HeLa cells was evaluated using fluorescence and laser scanning confocal microscopy (LSCM). **Eu.1** (250 μM) was incubated in HeLa cells for 4 hours, resulting in uptake of the Eu(iii) complex and predominant localisation to the mitochondria (*λ*_exc_ = 355 nm, *λ*_em_ = 605–720 nm), confirmed by colocalisation studies using MitoTracker Green (*λ*_exc_ = 488 nm, *λ*_em_ = 500–530 nm), which gave a Pearson's coefficient, *P* = 0.80 (Fig. 6A–C). The preferential distribution of **Eu.1** in the mitochondria augured well for the detection of endogenous ONOO^−^, because the precursor species, NO and O_2_˙^−^, react predominately in the mitochondria, and we have previously demonstrated that these reactive species do not interfere with the emission response of **Eu.1** (Fig. 4B).

A time-lapsed series of images revealed maximum uptake **Eu.1** in HeLa cells after 4 hours (Fig. S10A†). Imaging of **Eu.1** in the mitochondria was possible over extended time periods (up to 40 hours), during which time the brightness of the observed images did not vary significantly (±15%) and the cells appeared to be healthy and growing. Analysis of ICP-MS data showed that for 4 × 10^6^ HeLa cells incubated with **Eu.1** (250 μM), a given cell contained 26 μM (±10%) of **Eu.1** (Fig. S10C†), hence the intracellular concentration of the Eu(iii) probe reaches 10% of the loading buffer after 4 hours. Cytotoxicity studies were undertaken for **Eu.1** at 24 hours using image cytometry assays (involving DAPI and acridine orange stains), which showed an IC_50_ value of greater than 250 μM (Fig. S10B†). Therefore, it can be assumed that the Eu(iii) probe did not exhibit cytotoxicity during the timeframe of these imaging experiments.

### Imaging changes in peroxynitrite levels in living cells

Next, HeLa cells stained with **Eu.1** were incubated with the ONOO^−^ donor, SIN-1, for 30 minutes. This resulted in a significant (approx. 90%) decrease in emission intensity (Fig. 6E), indicating an increase in mitochondrial ONOO^−^ levels upon SIN-1 stimulation. It is unlikely that any ONOO^−^ generated in the cell culture medium will cross cell membranes;^3,41^ however, the ability of NO to penetrate cell membranes is known. So, the observed decrease in image brightness is consistent with SIN-1 mediated delivery of NO inside the cell, which combines with superoxide in the mitochondria to form ONOO^−^ spontaneously.^2^ In contrast, incubation of **Eu.1**-stained cells with SIN-1 and the superoxide scavenger, TEMPO, induced a significantly smaller (35%) decrease in luminescence compared with untreated cells (Fig. 6F), indicating reduced intracellular formation of ONOO^−^ compared with the SIN-1 treatment alone.

The Eu(iii) probe remained predominantly in the mitochondria during the time scale of the experiments. Further, ICP-MS analysis of cells treated with SIN-1 (and SIN-1 plus TEMPO), showed less than 10% variation in the concentration of accumulated Eu(iii), relative to untreated cells (Fig. S10C†). Therefore, the possibility that incubation with SIN-1 and/or TEMPO promotes efflux of **Eu.1** from cells can be ruled out on the timescale of the experiment.

To confirm that the increase in intracellular ONOO^−^ is responsible for the decrease in emission of **Eu.1**, a control experiment was undertaken using probe **Eu.Bn**, which lacks the reactive boronic acid moiety. LSCM images, following incubation of HeLa cells with **Eu.Bn** (250 μM), revealed a similar localisation to the mitochondria (Fig. S12A–C†), although a slightly slower rate of uptake was observed compared with **Eu.1** (maximum uptake at 18 hours, Fig. S11†). Crucially, upon treatment of **Eu.Bn**-stained HeLa cells with SIN-1, less than a 5% decrease in Eu(iii) emission intensity was observed, and the simultaneous addition of SIN-1 and TEMPO induced a negligible change in emission intensity (Fig. S12D–G†). Thus, **Eu.Bn** serves as an effective negative control probe, verifying that the SIN-1 induced change in luminescence of **Eu.1** corresponds to an increase in mitochondrial ONOO^−^ levels. Taken together, these live-cell imaging experiments demonstrate the powerful potential of **Eu.1** for visualising changes in ONOO^−^ concentration within the mitochondria of living cells.

### Time-resolved measurement of peroxynitrite in living cells treated with cold atmospheric plasma

To further demonstrate the biological applications of **Eu.1**, the probe was used to monitor dynamic changes in ONOO^−^ levels in different cell lines, following treatment with a commercially available cold atmospheric plasma device, kINPEN® MED, which is used in the clinic for skin wound treatment.^10^ Plasma generated NO and ONOO^−^ are proposed to interact with bacterial cell membranes, forming pores that promote further ingress of RONS. There are multiple mechanisms proposed for the intracellular formation of ONOO^−^, following plasma treatment.^47,48^ One mechanism involves plasma generated NO penetrating cell membranes and combining with O_2_˙^−^ in the mitochondria to form ONOO^−^. Therefore, we wished to test the ability of **Eu.1** to selectively visualise intracellular ONOO^−^, derived from RONS generated externally by the plasma.

First, the expected breakdown of **Eu.1** to **Eu.8HQ** upon treatment with ionizing plasma was confirmed by HR-TOF measurements of buffered aqueous samples containing **Eu.1** (Fig. S14†), reflecting the formation of ONOO^−^ in the treated liquid. Next, **Eu.1** (250 μM) was incubated with two different cell lines, THP and Jurkat cells, for 24 hours. The cells were centrifuged, washed with 100 mM PBS, and suspended in a glass bottom 96-well plate. Time-resolved luminescence spectra of **Eu.1** within the cells were recorded using a microplate reader (Fig. S13†).

**Fig. 7:**
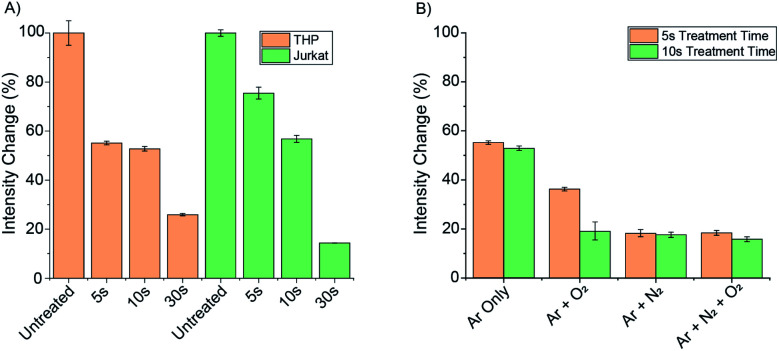
(A) Treatment of THP and Jurkat cell lines with the kINPen™ MED device . THP and Jurkat cells show significant loss of **Eu.1** signal following plasma treatment; (B) different gas mixtures induce different intensity responses of **Eu.1** in THP cells. Conditions: time-resolved emission (*λ*_ex_ = 321, *λ*_em_ = 670–720 nm, integration time 60–400 μs), 5 mM PBS, pH 7.4.

Samples of each cell line were then treated with cold atmospheric plasma using the kINPen® MED device for 5, 10 and 30 seconds (using an argon gas flow) and the change in time-resolved luminescence was recorded (Fig. 7A). For both cell lines, a 75–90% decrease in total emission intensity of **Eu.1** was observed after 30 seconds, corresponding to a significant increase in intracellular ONOO^−^. In THP cells, the emission intensity had decreased by 45% following 5 seconds treatment, whereas Jurkat cells showed a 25% decrease in luminescence under the same conditions, indicating a lower initial level of ONOO^−^ in Jurkat cells.

To investigate the effect of different plasma gases on the intracellular concentration of ONOO^−^, 0.5% oxygen and 0.5% nitrogen was added individually and in combination to the argon gas flow. THP cells incubated with **Eu.1** (250 μM), were then treated with the kINPen® MED device for 5 and 10 seconds using the different gas flows (Fig. 7B). The introduction of 0.5% N_2_ gas induced a substantial 80% decrease in emission intensity of **Eu.1** after 5 seconds, whereas only a 45% decrease in luminescence was observed when using argon gas alone. This correlates with a higher intracellular level of ONOO^−^ when nitrogen is used in the feed gas, consistent with a higher concentration of NO generated by the plasma. This corresponds perfectly with the observed modulation of NO generation by plasmas in pure argon and argon doped with molecular gases, respectively.^49,50^ Thus, **Eu.1** can be used to visualise elevated levels of ONOO^−^ in different cell lines following extracellular treatment with a plasma therapy device.

## Conclusions

We have developed a robust, water-soluble luminescent europium(iii) probe for the rapid and sensitive detection of ONOO^−^ in living cells. The probe displays excellent selectivity for ONOO^−^ over other RONS, and provides a change in luminescence that is directly proportional to ONOO^−^ concentration in the nanomolar to low micromolar range. The Eu(iii) probe offers advantages over classical organic fluorescent probes, including an instantaneous and long-lived luminescence signal, which enables time-resolved measurements of ONOO^−^ to be undertaken, avoiding the influence of autofluorescence in biological samples, thereby enhancing precision and signal-to-noise.

To demonstrate the utility of our probe, a plate reader-based assay was developed for the quantification of ONOO^−^ in human serum, with minimal interference from other reactive species, biological cations, anions and protein. The Eu(iii) probe was shown to permeate a range of different cell lines and localise preferentially to the mitochondria, allowing the presence of endogenous and/or externally induced ONOO^−^ to be monitored with sub-cellular resolution using fluorescence microscopy. Finally, the Eu(iii) probe was applied for the first time in monitoring elevated levels of ONOO^−^ in living cells, following treatment with a cold atmospheric plasma device standardised for clinical applications. The probe design features established in this work will inform the future development of emissive Eu(iii)/Tb(iii) probes, capable of measuring ONOO^−^ by providing a ratiometric signal that is intrinsically normalised, and thus independent of probe concentration.

In conclusion, the Eu(iii) probe can provide valuable insight into the spatial and temporal distribution of ONOO^−^ in cells, enabling a better understanding of its roles in cell biology and the mechanisms of transport of RONS into cells. This could facilitate the development of more effective therapies and devices, tailored to delivering specific levels of ONOO^−^ to targeted sites within cells and enabling safe doses of treatment to be determined.

## Conflicts of interest

There are no conflicts to declare.

## Supplementary Material

SC-011-C9SC06053G-s001

SC-011-C9SC06053G-s002

## References

[cit1] Graves D. B. (2012). J. Phys. D: Appl. Phys..

[cit2] Pacher P., Beckman J. S., Liaudet L. (2007). Physiol. Rev..

[cit3] Radi R. (2013). J. Biol. Chem..

[cit4] Bartesaghi S., Radi R. (2018). Redox Biol..

[cit5] Koppenol W. H. (1998). Quim. Nova.

[cit6] Spiteller G. (2006). Free Radical Biol. Med..

[cit7] Ferrer-Sueta G., Campolo N., Trujillo M., Bartesaghi S., Carballal S., Romero N., Alvarez B., Radi R. (2018). Chem. Rev..

[cit8] Manda G., Isvoranu G., Comanescu M. V., Manea A., Debelec Butuner B., Korkmaz K. S. (2015). Redox Biol..

[cit9] Keidar M. (2015). Plasma Sources Sci. Technol..

[cit10] Bekeschus S., Schmidt A., Weltmann K. D., von Woedtke T. (2016). Clin. Plasma Med..

[cit11] Bruggeman P. J., Kushner M. J., Locke B. R., Gardeniers J. G. E., Graham W. G., Graves D. B., Hofman-Caris R. C. H. M., Maric D., Reid J. P., Ceriani E., Fernandez Rivas D., Foster J. E., Garrick S. C., Gorbanev Y., Hamaguchi S., Iza F., Jablonowski H., Klimova E., Kolb J., Krcma F., Lukes P., MacHala Z., Marinov I., Mariotti D., Mededovic Thagard S., Minakata D., Neyts E. C., Pawlat J., Petrovic Z. L., Pflieger R., Reuter S., Schram D. C., Schröter S., Shiraiwa M., Tarabová B., Tsai P. A., Verlet J. R. R., Von Woedtke T., Wilson K. R., Yasui K., Zvereva G. (2016). Plasma Sources Sci. Technol..

[cit12] Lloyd G., Friedman G., Jafri S., Schultz G., Fridman A., Harding K. (2010). Plasma Processes Polym..

[cit13] Patange A., Boehm D., Ziuzina D., Cullen P. J., Gilmore B., Bourke P. (2019). Int. J. Food Microbiol..

[cit14] Hoffmann C., Berganza C., Zhang J. (2013). Med. Gas Res..

[cit15] Radi R. (2004). Proc. Natl. Acad. Sci. U. S. A..

[cit16] Sun X., Xu Q., Kim G., Flower S. E., Lowe J. P., Yoon J., Fossey J. S., Qian X., Bull S. D., James T. D. (2014). Chem. Sci..

[cit17] Chang M. C. Y., Pralle A., Isacoff E. Y., Chang C. J. (2004). J. Am. Chem. Soc..

[cit18] Bünzli J. C. G. (2014). J. Coord. Chem..

[cit19] Heffern M. C., Matosziuk L. M., Meade T. J. (2014). Chem. Rev..

[cit20] Szíjjártõ C., Pershagen E., Ilchenko N. O., Borbas K. E. (2013). Chem.–Eur. J..

[cit21] Hewitt S. H., Ali R., Mailhot R., Antonen C. R., Dodson C. A., Butler S. J. (2019). Chem. Sci..

[cit22] (b) Shuvaev S., Starck M. and Parker D., Chem.–Eur. J., 2017, 23, 9974 9989 28471496 10.1002/chem.201700567

[cit23] Butler S. J., Parker D. (2013). Chem. Soc. Rev..

[cit24] Mathieu E., Sipos A., Demeyere E., Phipps D., Sakaveli D., Borbas K. E. (2018). Chem. Commun..

[cit25] Wu J., Liu H., Yang Y., Wang H., Yang M. (2018). Opt. Mater..

[cit26] Song C., Ye Z., Wang G., Yuan J., Guan Y. (2010). Chem.–Eur. J..

[cit27] Poole R. A., Kielar F., Richardson S. L., Stenson P. A., Parker D. (2006). Chem. Commun..

[cit28] Law G. L., Parker D., Richardson S. L., Wong K. L. (2009). Dalton Trans..

[cit29] Sikora A., Zielonka J., Lopez M., Joseph J., Kalyanaraman B. (2009). Free Radical Biol. Med..

[cit30] Beeby A., Clarkson I. M., Dickins R. S., Faulkner S., Parker D., Royle L., de Sousa A. S., Williams J. A. G., Woods M. (1999). J. Chem. Soc., Perkin Trans. 2.

[cit31] Maffeo D., Williams J. A. G. (2003). Inorg. Chim. Acta.

[cit32] Farruggia G., Iotti S., Prodi L., Montalti M., Zaccheroni N., Savage P. B., Trapani V., Sale P., Wolf F. I. (2006). J. Am. Chem. Soc..

[cit33] Bozoklu G., Marchal C., Pécaut J., Imbert D., Mazzanti M. (2010). Dalton Trans..

[cit34] Bunzli J.-C. G., Claude P. (2005). Chem. Soc. Rev..

[cit35] Park S.-Y., Ghosh P., Park S. O., Lee Y. M., Kwak S. K., Kwon O.-H. (2016). RSC Adv..

[cit36] Cölle M., Dinnebier R. E., Brütting W. (2002). Chem. Commun..

[cit37] Bauer G. (2019). Redox Biol..

[cit38] Cheng D., Pan Y., Wang L., Zeng Z., Yuan L., Zhang X., Chang Y.-T. (2017). J. Am. Chem. Soc..

[cit39] Pattison D. I., Davies M. J. (2001). Chem. Res. Toxicol..

[cit40] Zielonka J., Sikora A., Hardy M., Joseph J., Dranka B. P., Kalyanaraman B. (2012). Chem. Res. Toxicol..

[cit41] Ferrer-Sueta G., Radi R. (2009). ACS Chem. Biol..

[cit42] Matata B. M., Galiñanes M. (2002). J. Biol. Chem..

[cit43] Lin A., Gorbanev Y., De Backer J., Van Loenhout J., Van Boxem W., Lemière F., Cos P., Dewilde S., Smits E., Bogaerts A. (2019). Adv. Sci..

[cit44] Girard F., Badets V., Blanc S., Gazeli K., Marlin L., Authier L., Svarnas P., Sojic N., Clément F., Arbault S. (2016). RSC Adv..

[cit45] Girard F., Peret M., Dumont N., Badets V., Blanc S., Gazeli K., Noël C., Belmonte T., Marlin L., Cambus J. P., Simon G., Sojic N., Held B., Arbault S., Clément F. (2018). Phys. Chem. Chem. Phys..

[cit46] Bauer G. (2019). Redox Biol..

[cit47] Tarabová B., Lukeš P., Hammer M. U., Jablonowski H., von Woedtke T., Reuter S., Machala Z. (2019). Phys. Chem. Chem. Phys..

[cit48] Lukes P., Dolezalova E., Sisrova I., Clupek M. (2014). Plasma Sources Sci. Technol..

[cit49] Schmidt-Bleker A., Bansemer R., Reuter S., Weltmann K.-D. (2016). Plasma Processes Polym..

[cit50] Jablonowski H., Schmidt-Bleker A., Weltmann K.-D., von Woedtke T., Wende K. (2018). Phys. Chem. Chem. Phys..

