# Supplementary material for: Time-resolved luminescence detection of peroxynitrite using a reactivity-based lanthanide probe
Source: Chem Sci. 2020 Feb 18;11(12):3164–70. doi: 10.1039/c9sc06053g (PMC8157329; doi:10.1039/c9sc06053g)
Supplement: SC-011-C9SC06053G-s001 [file SC-011-C9SC06053G-s001.pdf]

Electronic Supplementary Material (ESI) for Chemical Science.
This journal is © The Royal Society of Chemistry 2020

Supporting Information

Time-resolved luminescence detection of peroxynitrite using a reactivity-based lanthanide probe

Colum Breen,[a] Robert Pal,[b] Mark R. J. Elsegood,[a] Simon J. Teat,[c] Felipe Iza,[d] Kristian Wende,[e] Benjamin R. Buckley,[a] and Stephen J. Butler[a]*

[a]*Department of Chemistry, Loughborough University, Epinal Way, Loughborough, LE11 3TU, UK.*

[b]*Department of Chemistry, Durham University, South Road, Durham, DH1 3LE, UK.*

[c]*Advanced Light Source, Berkeley Lab., 1 Cyclotron Road, Berkeley, CA 94720, USA.*

[d]*Centre for Biological Engineering, Department of Mechanical, Electrical and Manufacturing Engineering, Loughborough University, Epinal Way, Loughborough, LE11 3TU, UK.*

[e]*Leibniz-Institute for Plasma Science and Technology, ZIK plasmatis, Felix-Hausdorff-Str.2, 17489 Greifswald, Germany.*

Experimental Section

1. General Procedures

NMR Spectroscopy

^1H, ^{13}C, COSY, HSQC and HMQC NMR spectra were recorded in the stated deuterated solvent on a JEOL–ECS-400 spectrometer (^1H at 399.78 MHz, ^{13}C at 100.53 MHz) a JEOL–ECS-500 spectrometer (^1H at 500.16 MHz, ^{13}C at 125.77 MHz), or a Bruker Advance Ultra-Shield 400 spectrometer (^1H at 400.13 MHz, ^{13}C at 100.62 MHz) at 293 K. Chemical shifts are expressed in ppm and are adjusted to the chemical shift of the residual NMR solvent resonances ($CDCl_3$: ^1H δ = 7.26 ppm, ^{13}C δ = 77.16 ppm, CD_3OD: ^1H δ = 3.31 ppm, ^{13}C δ = 49.00 ppm or D_2O: ^1H δ = 1.56 ppm). The coupling constants are expressed in Hz.

Mass Spectrometry

Liquid Chromatography Electrospray mass spectra were recorded on a Shimadzu LC-2020 system with controlled using LabSolutions, version 5.86 SP1 software. The system operates in positive or negative ion mode, with acetonitrile as the carrier solvent. The flow rate was maintained at 0.7 mL/min over a gradient of 5 to 95% acetonitrile (0.03% formic acid) in water (0.03% formic acid) for 10 minutes. High resolution mass spectra were recorded using a Thermofisher Q-Exactive Orbitrap mass spectrometer.

Chromatography

Column chromatography was performed using flash silica gel 60 (particle size 40–63 microns) purchased from Apollo Scientific. Thin layer chromatography (TLC) was performed on aluminium sheet silica gel plates with 0.2 mm thick silica gel 60 F_{254} using the stated mobile phase.

Preparative RP-HPLC was performed using a Waters 2489 UV/Visible detector performed at 254 nm, a Waters 1525 Binary HPLC pump controlled by the Waters Breeze 2 HPLC system software. Separation was achieved using a semi-preparative XBridge C18 (5 µm OBD 19 × 100 mm) column at a flow rate maintained at 17 mL/min. A solvent system composed of either water (0.05% formic acid) / acetonitrile (0.05% formic acid) or water (25 mM NH_4HCO_3) / acetonitrile was used over the stated linear gradient (usually 0 to 100% organic solvent over 10 minutes). Analytical RP-HPLC was performed using a XBridge C18 (5 µm 4.6 × 100 mm) column at a flow rate maintained at 2.0 mL/min using the stated gradient and solvents.

pH Measurements

Measurements of pH were carried out using a Jenway 3510 pH/mV meter with a Jenway combination electrode or a Jenway 3020 pH meter with an Aldrich glass combination pH electrode, both calibrated using buffer solutions of pH 4.00 ± 0.01, 7.00 ± 0.01 and 10.00 ± 0.01.

Optical Spectroscopy

Emission spectra were recorded on an SPEX Fluoromax luminescence spectrometer using dM3000 version 3.12 software. Emission spectra were obtained using either a 40 µL Hellma® Analytics quartz cuvette (Art no. 111-10-K-40) or a 1 mL Hellma® Analytics quartz cuvette (Art no. 101-10-K-40), with excitation at 321 nm and reading emission in the range 550 – 720 nm using an integration time of 0.5 seconds, increment of 1 nm and excitation and emission slits of 0.5 nm.

UV/Visable absorbance spectra were measured using a Shimadzu UV-1800 UV-spectrophotometer. Molar extinction coefficients were determined by first dissolving a known amount of Eu(III) complex in a known amount of solvent to give a bulk solution. Small volume additions were added to a known amount of buffer and absorbance spectra were recorded, slowly increasing absorbance until > 0.5 absorbance. Molar extinction coefficients, ε, were calculated in accordance with the Beer-Lambert law, by plotting absorbance, A, against complex concentration, c. Quantum yields were measured using **Eu.Bn** in water as a standard (Φ_{em}= 0.06).[1]

Lifetime measurements were performed using a Camlin Photonics luminescence spectrometer with FluoroSENS version 3.4.7.2024 software. Lifetime measurements were typically obtained by indirect excitation of the Eu(III) ion via the chromophore using a short pulse of light (at λ_{max}) followed by monitoring the integrated intensity of the light emitted at 615 nm during a fixed gate time, t_g, after a delay time, t_d. Measurements were made for a minimum of 20 delay times, covering 3 or more lifetimes. A gate time of 0.1 ms was used and the excitation and emission slits were set to 10 and 5 nm respectively. The obtained decay curves were plotted in Origin Labs 2019 (64-bit) ver. 9.6.0.172and fitted to the equation:

$$I = A_0 + A_1 e^{-kt}$$

Where:

I: intensity at time, t, following excitation; A_0: intensity when decay has ceased

A_1: pre-exponential factor; k: rate constant for the depopulation of the excited state.

Plate Reader

364 black well plates were used for plate reader-based analysis. Luminescence intensities were taken with excitation at 292 – 366 nm and emission using time-resolved measurements at 615 – 625 nm using an integration time of 60 – 400 μs. For plate-based analysis of cells, Tecan M200 Infinite plate reader, operated with iControl software was used. An excitation of 321 nm was used, and emission was collected at 550 – 720 nm, with a 60 – 400 μs time-gate.

Cell Culture

A detailed investigation of the cellular behavior of each Eu(III) complex was conducted using HeLa (humane cervical cancer) cell line using laser scanning confocal microscopy. Cells were maintained in exponential growth as monolayers in F-12/DMEM (Dulbecco's Modified Eagle Medium) 1:1 that was supplemented with 10% foetal bovine serum (FBS). Cells were grown in 75 cm^2 plastic culture flasks, with no prior surface treatment. Cultures were incubated at 37°C, 20% average humidity and 5% (v/v) CO_2. Cells were harvested by treatment with 0.25% (v/v) trypsin solution for 5 minutes at 37ºC. Cell suspensions were pelleted by centrifugation at 1000 rpm for 3 minutes, and were re-suspended by repeated aspiration with a sterile plastic pipette. Microscopy Cells were seeded in 12-well plates on 13 mm 0.170 mm thick standard glass cover-slips and allowed to grow to 40% – 60% confluence, at 37ºC in 5% CO_2. At this stage, the medium was replaced and cells were treated with complexes and co-stains as appropriate. For imaging DMEM media (10% FBS) lacking phenol red (live cell media) was used from this point onwards. Following incubation, the cover-slips were washed with live cell media,

mounted on slides and the edges sealed with colourless, quick-dry nail varnish to prevent drying out of the sample.

Cell toxicity measurements were run using a ChemoMetec A/S NucleoCounter3000-Flexicyte instrument with Via1-cassette cell viability cartridge (using the cell stain Acridine Orange for cell detection, and the nucleic acid stain DAPI for detecting non-viable cells). The experiments were carried out in triplicate. In cellular uptake studies, cells were seeded in 6-well plates and allowed to grow to 80% – 100% confluence, at 37ºC in 5% CO_2. At this stage, the medium was replaced with media containing Eu(III) complexes as detailed above and total cellular europium was determined using inductively coupled plasma mass spectrometry (ICP-MS), by Dr. C. Ottley in the Department of Earth Sciences at Durham University.

Steady state fluorescence microscopy

Steady state fluorescence images were recorded using a PhMoNa enhanced Leica SP5 II LSCM confocal microscope equipped with a HCX PL APO 63x/1.40 NA LambdaBlue Oil immersion objective. Data were collected using 2.5x digital magnification at 100 Hz/line scan speed (2 line average, bidirectional scanning) at 355 nm (3rd harmonic NdYAG laser) with 3 mW laser power (80 nJ/voxel). In order to achieve excitation with maximal probe emission (605 – 720 nm), the microscope was equipped with a triple channel imaging detector, comprising a conventional PMT systems and two HyD hybrid avalanche photodiode detector. The latter part of the detection system, when operated in the BrightRed mode, is capable of improving imaging sensitivity by 25%, reducing signal to noise by a factor of 5. Frame size was determined at 1024 x 1024 pixel, with 0.6 airy disc unit determining the applied pinhole diameter rendering on voxel to be corresponding to 62 x 62 nm (frame size 96 x 96 μm) with a section thickness of 380 nm. A HeNe (488 nm, 2 mW) laser was used when commercially available organelle-specific stains (e.g. MitoTrackerRed™; 500 – 530 nm) were used to corroborate cellular compartmentalization.

2. Preparation of reactive species

A known concentration stock solution of reactive species was prepared in water, pH 7.4. A known volume of each was added to 1 mL of the Eu(III) complex (25 μM, 100 mM PBS, pH 7.4) and an emission spectrum was taken every 2 minutes, for 30 minutes. Appropriate ratios of either the ΔJ = 2/ΔJ = 1 (605 – 630 nm / 585 – 600 nm) or ΔJ = 2/ΔJ = 0 (605 – 630 nm / 575 – 583 nm) were taken and changes in intensity were compared to the starting intensity. For NO and $^1O_2$, analysis every 2 minutes was not possible due to the generation method, thus a spectrum was taken at 0, 15 and 30 minutes. Changes in intensity were compared with initial intensity. Due to the short lifetime, $OH^{\bullet}$ and $ROO^{\bullet}$ radicals were generated *in situ*.

OCl^-

OCl^- stock solution was prepared from 5% active chlorine solution purchased from Sigma Aldrich in deionised deoxygenated water. The stock solution was used for subsequent dilutions

$ONOO^-$

A stock solution was purchased from Cayman Chemicals (in a 0.3 M NaOH solution) and its concentration was calculated using the extinction coefficient 1670 M^{-1} cm^{-1}.[2] The stock solution was used for subsequent dilutions.

H_2O_2

A stock solution was purchased from Sigma Aldrich (30% H_2O_2/H_2O, v/v%). Stock solutions were prepared with 30% H_2O_2 in water using deionised deoxygenated water. The stock solution was used for subsequent dilutions.

$OH^{\bullet -}$

Hydroxyl radical was generated by the Fenton reaction. To prepare $OH^{\bullet}$ solution, hydrogen peroxide (H_2O_2, 10 equivalents) was added to $FeCl_2$ in deionised deoxygenated water. The stock solution was used for subsequent dilutions .

$O_2^{\bullet -}$

Superoxide was generated from KO_2. KO_2 and 18-crown-6 ether (2.5 equivalents) were dissolved in DMSO to afford a 0.25 M solution. The stock solution was used for subsequent dilutions.

NO

Nitric oxide (NO) was prepared by treating sodium nitrite (25 mg, 360 μmol) with acidified ferrous sulphate (1.8 M H_2SO_4 and 1.2 M Fe^{2+}). The subsequent gas was bubbled through a solution containing **Eu.1.** Diethylamine NONOate sodium salt (DEANO) was also used to prepare a stock of NO. The stock solution was used for subsequent dilutions.

$^1O_2$

Singlet oxygen was generated *in situ* from the photosensitisation of rose bengal. Rose bengal (25 μM) was added to **Eu.1** solution (25 μM), and a full screen was conducted (scan every 2 minutes, over 30

minutes). No emission changes were observed. **Eu.1** + rose bengal solution was pumped through a photoreactor for 10 mins, three times (λ_{ir} = 550 nm), and the emission intensity was analysed.

3. Compound Synthesis and Characterisation

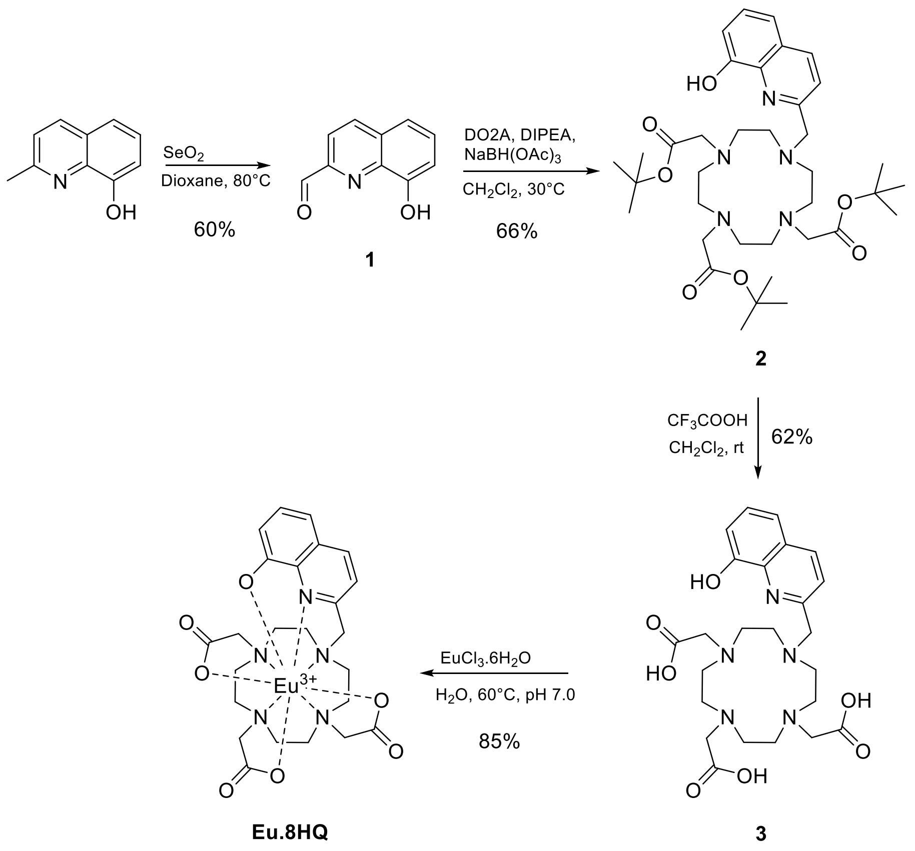

Scheme S1. Synthesis of complex **Eu.8HQ.**

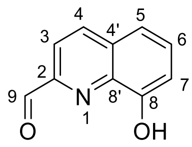

1

To a solution of commercially available 2-methylquinolin-8-ol (1.00 g, 6.28 mmol) in 1,4-dioxane (15 mL), was added selenium dioxide (0.871 g, 7.85 mmol). The bright yellow reaction mixture was stirred at 80°C for 24 hours. The reaction mixture was filtered over celite and washed with ethyl acetate (3 × 10 mL). The solvent was removed under reduced pressure. A dark orange solid was collected as the crude product, which was purified by column chromatography (silica gel; 20% ethyl acetate/hexane) to give the desired compound **1** as a yellow solid (60%, 0.657 g). ^{1}H NMR (400 MHz, $CDCl_3$) δ 10.21 (s, 1H, H^9), 8.31 (d, ^{3}J = 8.5 Hz, 1H, H^4), 8.05 (d, ^{3}J = 8.5 Hz, 1H, H^3), 7.62 (t, ^{3}J = 8.0 Hz, 1H, H^6), 7.42 (d, ^{3}J = 8.0 Hz, 1H, H^5), 7.28 (d, ^{3}J = 8.0 Hz, 1H, H^7), 3.71 (s, 1H, OH). ^{13}C NMR (100 MHz, $CDCl_3$) δ 192.9 (C^9), 153.1 (C^2), 150.2 (C^8), 137.9 ($C^{8'}$), 137.6 (C^3), 131.1 (C^6), 130.5 ($C^{4'}$), 118.2 (C^5), 118.1 (C^4), 111.3 (C^7). R_f = 0.35 (20% ethyl acetate/hexane). The spectroscopic data were in agreement with those reported previously. [3]

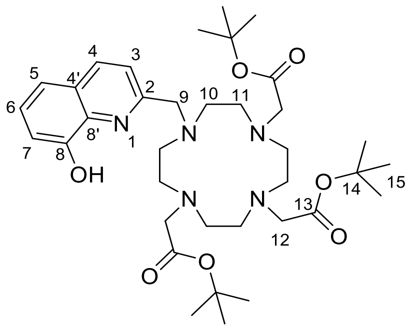

2

To a solution of 8-hydroxyquinoline-2-carboxaldehyde **1** (0.200 g, 1.15 mmol), in dichloromethane (15 mL), was added tri-*tert*-butyl 2,2',2''-(1,4,7,10-tetraazacyclododecane-1,4,7-triyl)triacetate (0.500 g, 0.969 mmol), and *N,N*-diisopropylethylamine (0.279 mL, 1.44 mmol). The mixture was stirred at room temperature for 10 minutes. Sodium triacetoxyborohydride (0.306 g, 1.44 mmol) was added and the bright yellow cloudy reaction mixture was stirred at 30°C for 72 hours. The reaction mixture was partitioned between ammonium bicarbonate solution (10 mL) and dichloromethane (20 mL). The aqueous layer was extracted with dichloromethane (3 x 10 mL). The organic layers were combined and dried over $MgSO_4$. The organic solvent was removed under reduced pressure and a yellow oil was collected as the crude product, which was purified by column chromatography (silica gel; neat dichloromethane to 5% v/v methanol/dichloromethane) to give the desired compound **2** as a yellow oil (66%, 0.435 g). ^{1}H NMR (400 MHz, $CDCl_3$) δ 8.25 (d, ^3J = 8.5 Hz, 1H, H^4), 7.86 (d, ^3J = 7.5 Hz, 1H, H^5), 7.82 (d, ^3J = 8.5 Hz, 1H, H^3), 7.57 (m, 2H, $H^{6,7}$), 4.04 (s, 2H, H^9), 3.43 – 2.12 (m, 22H, $H^{10, 11, 12}$), 1.50 – 1.36 (m, 27H, H^{15}). ^{13}C NMR (100 MHz, $CDCl_3$) 173.7, 172.7, 170.7, 158.9, 146.1, 140.3, 136.8, 128.6, 128.2, 126.2, 124.1, 121.1, 119.4, 82.9, 82.6, 59.6, 57.5, 56.6, 56.0, 51.3, 49.5, 47.3, 28.2, 28.0, 27.9. R_f = 0.4 (2% CH_3OH/CH_2Cl_2), HR-MS ESI: 694.4142 $[M+Na]^+$ $[C_{36}H_{57}O_7N_5Na]^+$ requires 694.4150.

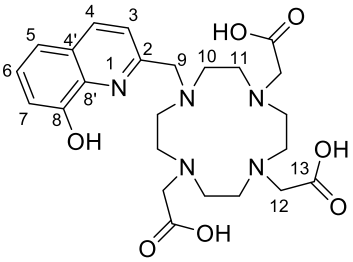

3

To a solution of **2** (100 mg, 0.150 mmol) in dichloromethane (8 mL), was added trifluoroacetic acid (4 mL). The bright yellow reaction mixture was stirred at room temperature for 96 hours. The trifluoroacetic acid was co-evaporated with CH_2Cl_2 (5 × 10 mL) and a yellow oil was collected as the crude product. The crude material was purified by preparative RP-HPLC [gradient: 0 − 100% CH_3OH with 0.1 % formic acid, over 10 minutes at 17 mL min^{-1}; T_R = 4.78 minutes] to give deprotected ligand **3** as a yellow solid (62%, 47 mg). ^1H NMR (400 MHz, D_2O) δ 8.38 (d, ^3J = 8.0 Hz, 1H, H^4), 7.80 (d, ^3J = 8.0 Hz, 1H, H^3), 7.47 – 7.44 (m, 2H, $H^{5,\,6}$), 7.25 – 7.19 (m, 1H, H^7), 4.05 (s, 2H, H^9), 3.60 – 2.80 (m, 22H, $H^{10,\,11,\,12}$), O-H signals not observed. ^{13}C NMR (100 MHz, D_2O) δ 176.9 (C^{13}), 169.6 (C^2), 149.5 (C^8), 142.6 (C^4), 140.5 ($C^{8'}$), 135.6 ($C^{4'}$), 129.2 (C^6), 128.8 (C^5), 122.8 (C^3), 120.2 (C^6), 116.2 (C^7), 57.0 (C^9), 56.3 (C^{cyclen}), 54.6 (C^{cyclen}), 51.6 (C^{cyclen}), 50.4 (C^{cyclen}), 48.2 (C^{cyclen}). HR-MS ESI: 504.2440 $[M+H]^+$ $[C_{24}H_{34}O_7N_5]^+$ requires 504.2452.

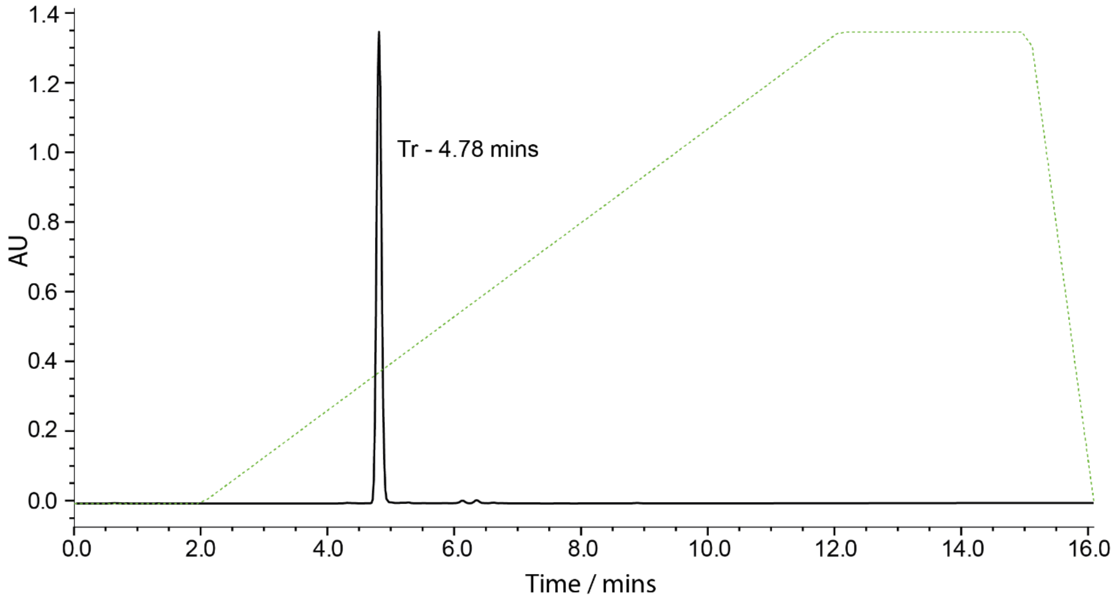

Analytical RP-HPLC trace of ligand **3** [gradient: 0 − 100% CH_3OH with 0.1 % formic acid, over 10 minutes at 17 mL min^{-1}; T_R = 4.78 minutes].

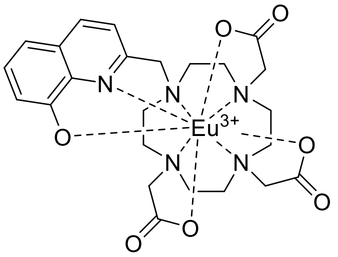

Eu.8HQ

Deprotected ligand **3** (10 mg, 20 μmol) was dissolved in H_2O (1 mL) and the pH adjusted to pH 7.0 using 0.3 M NaOH solution. $EuCl_3.6H_2O$ (9 mg, 24 μmol) was added and the pH was readjusted to 7.0 using dilute NaOH solution. The yellow reaction mixture was stirred at 60 °C for 24 hours. The reaction mixture was purified by preparative RP-HPLC [gradient: 0 − 100% CH_3CN in 50 mM NH_4HCO_3, over 10 minutes at 17 mL min^{-1}; T_R = 5.32 minutes] to give **Eu.8HQ** as a yellow solid (85%, 10 mg). HR-MS ESI: 676.1217 $[M+Na]^+$ $[C_{24}H_{30}EuNaO_7N_5]^+$ requires 676.1233. λ_{max} = 386 nm, ε = 2100 M^{-1} s^{-1}. ^1H NMR (400 MHz, D_2O, spectral range of 220 ppm (+120 to -100 ppm)), δ 29.10 (br), 17.70 (br), 7.25 (s), 5.69 (br), 5.12 (br), -4.95 (br), -6.68 (br), -14.56 (br).

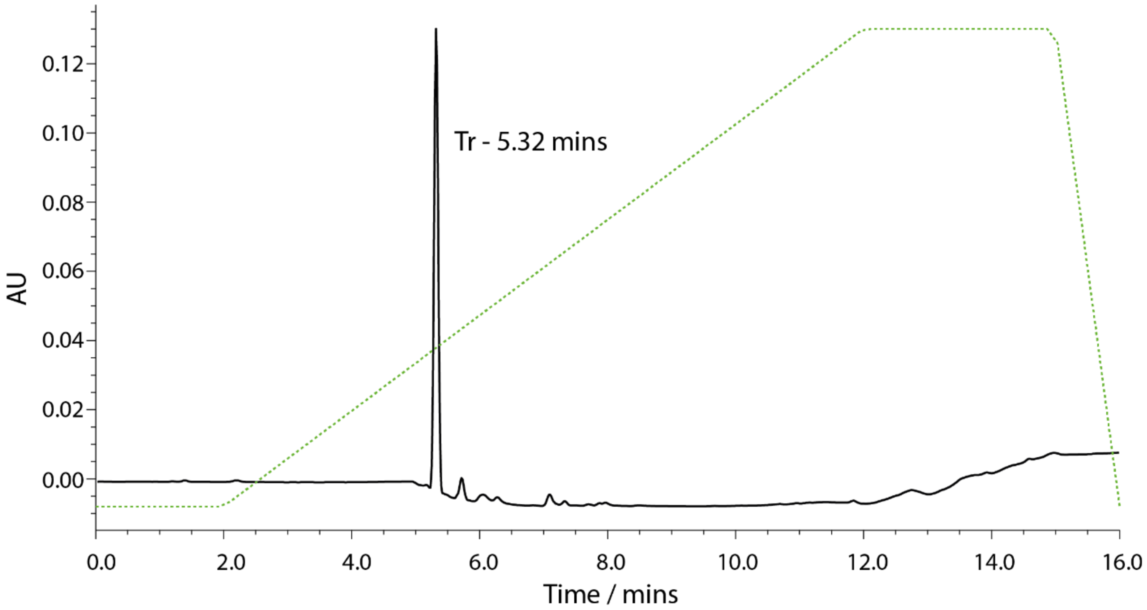

Analytical RP-HPLC trace of **Eu.8HQ** [gradient: 0 − 100% CH_3CN in 50 mM NH_4HCO_3, over 10 minutes at 17 mL min^{-1}; T_R = 5.32 minutes].

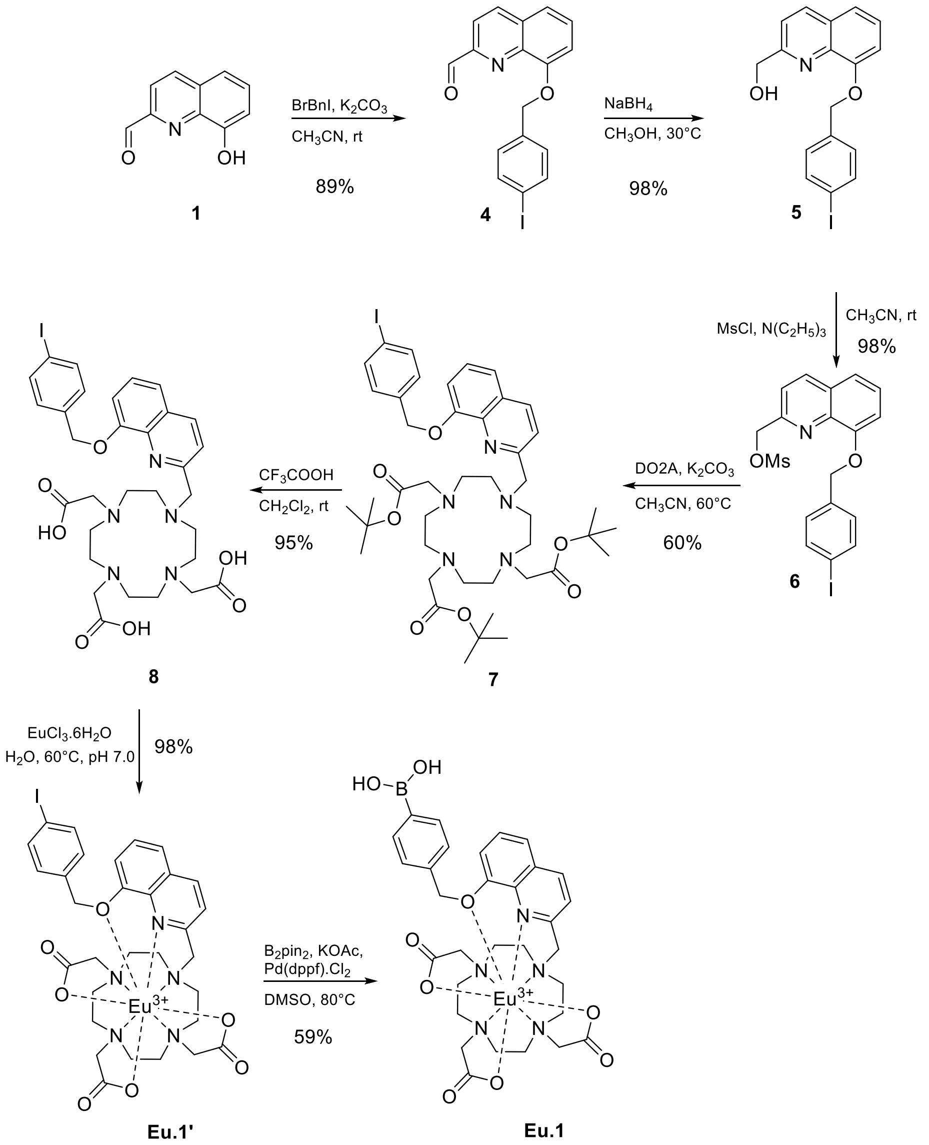

Scheme S2. Synthesis of complex **Eu.1**

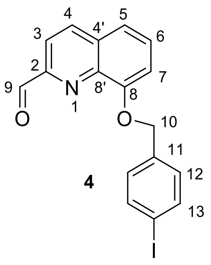

4

To a solution of **1** (0.500 g, 2.89 mmol) and potassium carbonate (1.00 g, 7.38 mmol) in anhydrous acetonitrile (15 mL) was added 4-iodobenzyl bromide (0.876 g, 2.95 mmol). The reaction mixture was stirred at room temperature for 24 hours. The resulting orange solution was centrifugated at 120 rpm for 3 minutes. The solution was decanted and the solid pellet was washed twice with CH_2Cl_2 (3 x 15 mL). The solvent was evaporated under reduced pressure and the resulting residue was partitioned between CH_2Cl_2 and saturated aqueous sodium chloride. The aqueous phase was extracted with CH_2Cl_2 (3 x 10 mL). The aqueous layer was washed with CH_2Cl_2 and the combined organic phases were dried over $MgSO_4$, filtered and concentrated under reduced pressure. The crude product was purified by column chromatography (silica gel; 20% v/v ethyl acetate/petroleum ether to 60% v/v ethyl acetate/petroleum ether) to give the desired compound **4** as a pink solid (89%, 1.01 g). ^{1}H NMR (400 MHz, $CDCl_3$) δ 10.31 (s, 1H, H^9) δ 8.29 (d, ^3J = 8.5 Hz, 1H, H^4), 8.08 (d, ^3J = 8.5 Hz, 1H, H^3), 7.73 (d, ^3J = 8.0 Hz, 2H, H^{13}), 7.67(d, ^3J = 8.0 Hz, 1H, H^5), 7.54 (t, ^3J = 8.0 Hz, 1H, H^6), 7.48 (d, ^3J = 8.0 Hz, 1H, H^3), 7.30 (d, ^3J = 8.0 Hz, 2H, H^{12}), 7.11 (d, ^3J = 8.0, 1H, H^7), 5.30 (s, 2H, H^{10}). ^{13}C-NMR (100 MHz, $CDCl_3$) δ 193.9 (C^9), 154.9 (C^8), 151.7 (C^2), 140.29 ($C^{8'}$), 137.9 ($C^{14, 15}$), 137.5 (C^4), 138.0 (C^5), 130.1 (C^6), 129.0 ($C^{12, 13}$), 126.4 ($C^{4'}$), 120.3 (C^7), 118.1 (C^3), 111.2 (C^7), 93.6 (C^{14}), 70.2 (C^{10}). R_f = 0.2 (25% ethyl acetate/hexane). HR-MS ESI: 389.9974 $[M+H]^+$ $[C_{17}H_{13}INO_2]^+$ requires 389.9985.

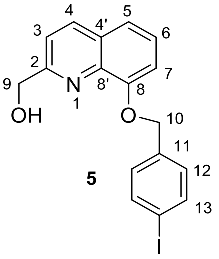

To a solution of aldehyde **4** (0.500 g, 1.28 mmol) in methanol (20 mL), was added sodium borohydride (61 mg, 1.61 mmol). The pale yellow reaction mixture was stirred at 30°C for 24 hours. The solvent was removed under reduced pressure and the resulting residue was partitioned between CH_2Cl_2 (20 mL) and saturated aqueous sodium chloride (10 mL). The aqueous layer was extracted with CH_2Cl_2 (3 × 20 mL) and the organic layers were combined, dried over $MgSO_4$ and concentrated under reduced pressure to give the desired alcohol **5** as a white solid (98%, 0.492 g). ^{1}H NMR (400 MHz, $CDCl_3$) δ 8.13 (d, ^3J = 8.5 Hz, 1H, H^4), 7.72 (d, ^3J = 8.0 Hz, 2H, H^{13}), 7.67 (d, ^3J = 8.0 Hz, 1H, H^5), 7.44 – 7.41 (m, 2H, $H^{6,\,5}$), 7.35 (d, ^3J = 8.0 Hz, 1H, H^3), 7.29 (d, ^3J = 8.0 Hz, 2H, H^{12}), 7.13 (d, ^3J = 8.0, 1H, H^7), 5.31 (s, 2H, H^{10}), 4.95 (s, 2H, H^9), 4.99 (br, 1H, OH). R_f = 0.35 (50% ethyl acetate/hexane). HR-MS ESI: 392.0135 $[M+H]^+$ $[C_{17}H_{15}INO_2]^+$ requires 392.0142.

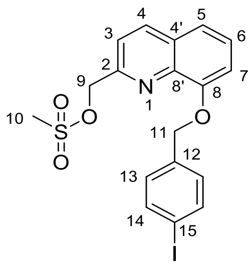

6

To a solution of alcohol **5** (0.100 g, 0.260 μmol) and triethylamine (39 mg, 55 μl, 0.38 μmol) in anhydrous acetonitrile (5 mL), was added methanesulfonyl chloride (32 mg, 22 μl, 0.28 μmol). The orange reaction mixture was stirred at room temperature for 3 hours. The solvent was removed under reduced pressure and the residue was partitioned between CH_2Cl_2 (15 mL) and saturated aqueous sodium chloride (5 mL). The aqueous layer was extracted with CH_2Cl_2 (3 × 10 mL) and the organic layers were combined, dried over $MgSO_4$ and concentrated under reduced pressure to give mesylate ester **6** as a bright yellow oil (98%, 0.120 g). ^{1}H NMR (400 MHz, CD_3OD) δ 8.16 (d, ^3J = 8.5 Hz, 1H, H^3), 7.58 (d, ^3J = 8.5 Hz, 1H, H^4), 7.45 (t, ^3J = 8.0 Hz, 1H, H^5), 7.37 (d, ^3J = 8.0 Hz, 1H, H^6), 7.04 (d, ^3J = 8.0 Hz, 1H, H^7), 5.52 (s, 2H, H^9), 4.03 (s, 3H, H^{10}), 3.10 (s, 2H, H^{11}). R_f = 0.25 (50% ethyl acetate/hexane). LR-MS ESI: 469.8 $[M+H]^+$ $[C_{18}H_{17}INO_4S]^+$ requires 469.3.

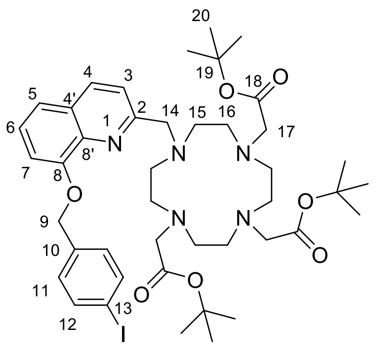

7

To a solution tri-*tert*-butyl 2,2',2''-(1,4,7,10-tetraazacyclododecane-1,4,7-triyl)triacetate (99 mg, 0.194 mmol) and potassium carbonate (80 mg, 0.58 mmol) in dry acetonitrile (10 mL) was added mesylate ester **6** (0.100 g, 0.213 mmol). The pale-yellow solution was stirred at 60°C for 24 hours, and the solution was centrifuged at 1500 rpm for 3 minutes. The organic layer was removed, and the pellet was washed with CH_2Cl_2 (2 x 10 mL). The organic layers were combined and the solvent was removed under reduced pressure. The crude product was purified by column chromatography (silica gel; 9:1 v/v% CH_2Cl_2/methanol) to give the desired compound **7** as a pale yellow solid (60%, 100 mg). ^{1}H NMR (400 MHz, CD_3OD) δ 8.09 (d, ^3J = 8.5 Hz, 1H, H^3), 7.66 (d, ^3J = 8.2 Hz, 2H, H^{12}), 7.59 (d, ^3J = 8.5 Hz, 1H, H^3), 7.38 – 7.30 (m, 9H, $H^{5,7}$), 7.20 (d, ^3J = 8.0 Hz, 2H, H^{11}), 6.95 (dd, ^3J = 7.0 Hz, ^4J = 2.0 Hz, 1H, H^6), 5.36 (s, 2H, H^9), 4.05 – 3.74 (m, 2H, H^{14}), 3.63 – 1.87 (m, 22H, H^{cyclen}), 1.39 – 1.29 (m, 27H, H^{20}). ^{13}C NMR (100 MHz, $CDCl_3$) δ 172.9 (C^{18}), 172.8 ($C^{18'}$) , 157.7 (C^2), 153.6 (C^8), 139.9 ($C^{8'}$), 137.8 (C^{12}), 136.8 (C^4), 128.7 (C^{11}), 126.5 (C^6), 122.6 (C^3), 120.4 (C^5), 111.4 (C^7), 93.2 (C^{13}), 70.3 (C^{14}), 56.5 (C^{14}), 50.9 (br, C^{cyclen}), 28.1 (C^{cyclen}). HR-MS ESI: 888.3752 $[M+H]^+$ $[C_{43}H_{63}IN_5O_7]^+$ requires 888.3767.

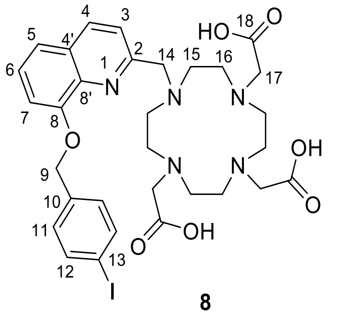

8

To a solution of the protected macrocyclic ligand **7** (25 mg, 28 μmol) in dichloromethane (8 mL), was added trifluoroacetic acid (4 mL). The brown reaction mixture was stirred at room temperature for 24 hours and then the trifluoroacetic acid was co-evaporated with CH_2Cl_2 (5 × 10 mL). A brown oil was collected as the crude product, which was purified by preparative RP-HPLC [gradient: 0 − 100% acetonitrile in 25 mM NH_4HCO_3, over 15 minutes at 17 mL min^{-1}; T_R = 10.5 minutes] to give the deprotected ligand **8** as a white solid (95%, 20 mg). ^{1}H-NMR (400 MHz, D_2O) δ 8.54 (s, br, 1H, H^4), 7.96 (s, br, 1H, H^5), 7.57 (d, ^3J = 7.5 Hz, 2H, H^{11}), 7.48 (s, 2H, $H^{6, 7}$), *7.27* (s, 1H, H^3), 7.13 (d, ^3J = 7.5 Hz, 2H, H^{12}), 5.21 (s, 2H, H^9), 4.16 (2H, H^{14}), 3.69 – 2.60 (22H, H^{cyclen}). ^{13}C-NMR (100 MHz, D_2O) δ 168.8 (C^{17}), 163.0 (C^2), 154.6 (C^8), 148.7 ($C^{8'}$), 146.5 (C^{10}), 137.5 (C^{12}), 135.3 (C^4), 130.3 (C^{11}), 129.8 ($C^{4'}$), 129.1 (C^6), 123.0 (C^3), 120.8 (C^5), 114.6 (C^7), 93.8 (C^{13}), 70.8 (C^9), 56.3 (C^{14}) 56.2 – 46.3 (C^{cyclen}). HR-MS ESI: 720.1883 $[M+H]^+$ $[C_{31}H_{39}IN_5O_7]^+$ requires 720.1889.

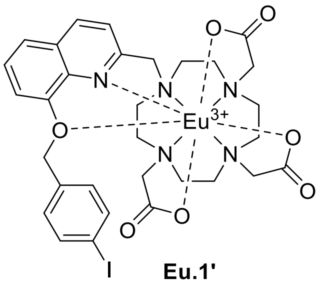

Eu.1'

Deprotected ligand **8** (10 mg, 14 µmol) was dissolved in H_2O (10 mL) and the pH adjusted to pH 7.5 using 0.3 M NaOH solution. $EuCl_3.6H_2O$ (6 mg, 17 µmol) was added and the pH was readjusted to 7.5 using 0.3 M NaOH solution. The yellow reaction mixture was stirred at 60°C for 24 hours. The crude reaction mixture was purified by preparative RP-HPLC [gradient: 0 − 100% acetonitrile in 25 mM NH_4HCO_3, over 10 minutes at 17 mL min^{-1}; T_R = 6.75 minutes] to give **Eu.1'** as a white solid (98%, 12 mg). ^1H NMR δ (500 MHz, D_2O, spectral range of 220 ppm (+120 to -100 ppm)), 31.37 (s), 18.13 (br), 15.43 (br), 10.72 (s), 9.84 (s), 8.45 - 7.50 (m), 4.61(s), 3.28 (s), 2.62 (s), 1.77 (s), 1.35 – 1.10 (m), 0.07 (s), -3.04 (br), -6.16 (br), -9.48 (br), -13.76 (br). HR-MS ESI: 870.0852 $[M+H]^+$ $[C_{31}H_{36}N_5O_7IEu]^+$ requires 870.0866. Extinction coefficient (ε) = 3400 M^{-1} cm^{-1}, τ_{H_2O} = 0.51 ms, τ_{D_2O} = 0.58 ms, q = 0.0, Φ = 4%.

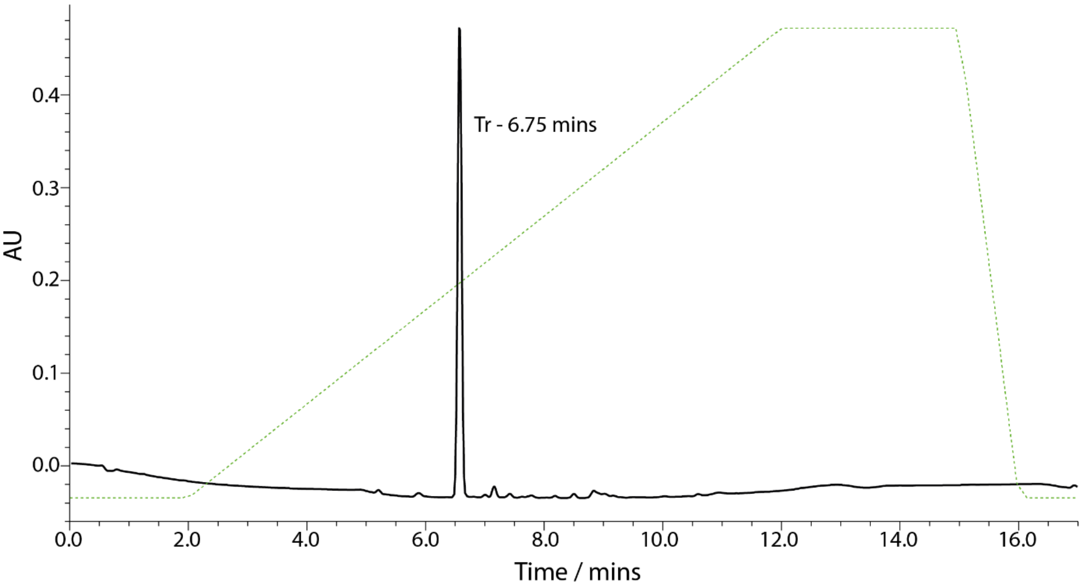

Analytical RP-HPLC trace of **Eu.1'** [gradient: 0 − 100% acetonitrile in 25 mM NH_4HCO_3, over 10 minutes at 17 mL min^{-1}; T_R = 6.75 minutes].

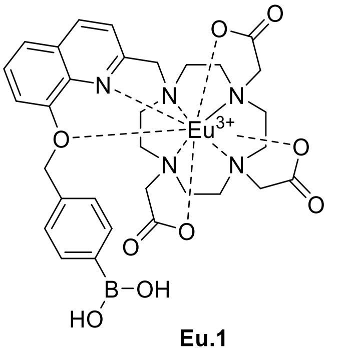

Eu.1

Eu.1’ (10 mg, 12 μmol) was dissolved in DMSO (2 mL) and potassium acetate (6 mg, 60 μmol) and bis(pinacolato)diboron (9 mg, 36 μmol) was added. The mixture was degassed by three freeze-pump-thaw cycles prior to the addition of [1,1′-bis(diphenylphosphino)ferrocene]dichloropalladium(II) (2 mg, 0.2 μmol). The reaction mixture was stirred at 80°C for 24 hours under an argon atmosphere. The solution was diluted with 100 mL of deionised H_2O and the solvent was removed by freeze drying. The resulting yellow solid was purified by preparative RP-HPLC [gradient: 0 − 100% acetonitrile in 0.05% v/v formic acid, over 10 minutes at 17 mL min^{-1}; T_R = 5.50 minutes] to give **Eu.1** as a white solid (59%, 4 mg). ^{1}H NMR δ (400 MHz, D_2O, Spectral range of 220 ppm (+120 to -100 ppm)) 27.90 (br), 25.49 (br), 20.52 (br), 17.75 (br), 16.25 (br), 12.44 (br), 10.29 (br), 9.09 (s), 8.36 (m), 7.58 (s), 7.21 (s), 6.81 (s), 2.63 (s), 2.13 (s), 1.63 (m), 1.13 (m), -3.69 (br), -4.53 (br), -8.59 (br), -13.62 (br), -17.75 (br), -20.33 (br). HR-MS ESI: 788.1955 $[M+H]^+$ $[C_{31}H_{38}N_5O_9BEu]^+$ requires 788.1969. Extinction coefficient (ε) = 2900 M^{-1} cm^{-1}, τ_{H_2O} = 0.52 ms, τ_{D_2O} = 0.66 ms, q = 0.2, Φ = 9%.

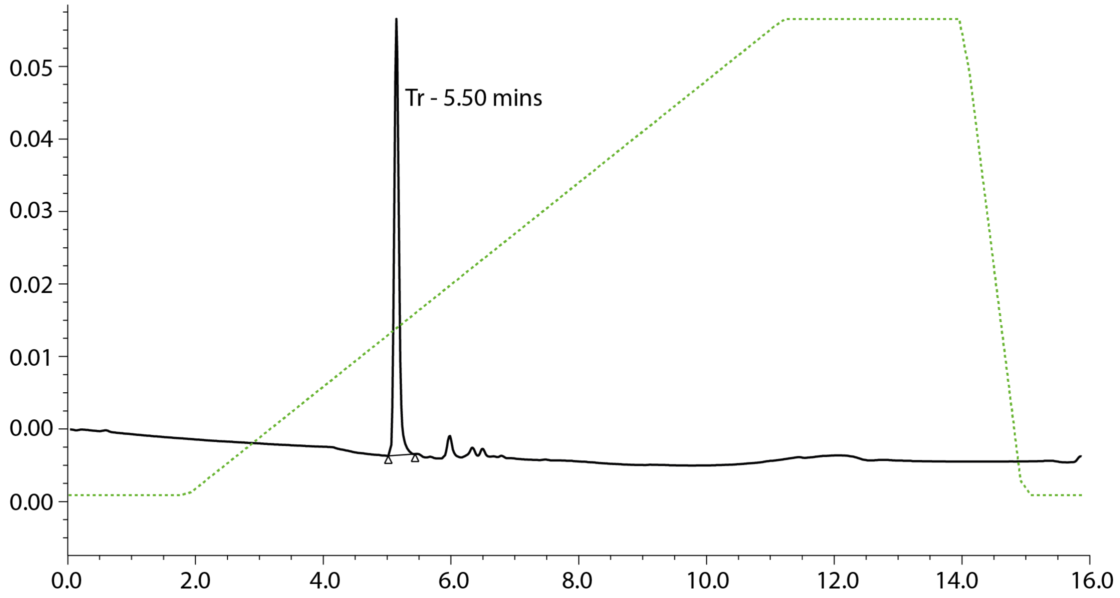

Analytical RP-HPLC trace of **Eu.1** [gradient: 0 − 100% acetonitrile in 0.05% v/v formic acid, over 10 minutes at 17 mL min^{-1}; T_R = 5.50 minutes]

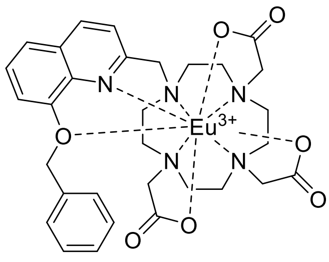

Eu.Bn

Eu.1' (10 mg, 12 μmol) was dissolved in DMSO (2 mL), H_2O (0.1 mL) and potassium acetate (6 mg, 60 μmol). The mixture was degassed by three freeze-pump-thaw cycles prior to the addition of [1,1′-bis(diphenylphosphino)ferrocene]dichloropalladium(II) (2 mg, 0.2 μmol) being added. The reaction mixture was stirred at 80°C for 24 hours under an atmosphere of argon. The solution was diluted with 100 mL of deionised H_2O and the solvent was removed by freeze drying. The resulting yellow solid was purified by preparative RP-HPLC [gradient: 0 − 100% acetonitrile in 0.05% v/v formic acid, over 10 minutes at 17 mL min^{-1}; T_R = 5.75 minutes] to give **Eu.Bn** as a pale yellow solid (58%, 5 mg). ^1H NMR δ (400 MHz, D_2O, spectral range of 220 ppm (+120 to -100 ppm)) 31.42 (br), 17.98 (br), 15.36 (br), 10.72 (s), 9.87 (br), 7.94 (br), 4.61 (s), 3.28 (s), 1.78 (s), -3.07 (s), -6.27 (br), -9.50 (br), -13.84 (br). HR-MS ESI: 744.1905 $[M+H]^+$ $[C_{31}H_{37}N_5O_7Eu]^+$ requires 744.1900. Extinction coefficient (ε) = 2600 M^{-1} cm^{-1}, τ_{H_2O} = 0.58 ms, τ_{D_2O} = 0.72 ms, q = 0.1, Φ = 6%.

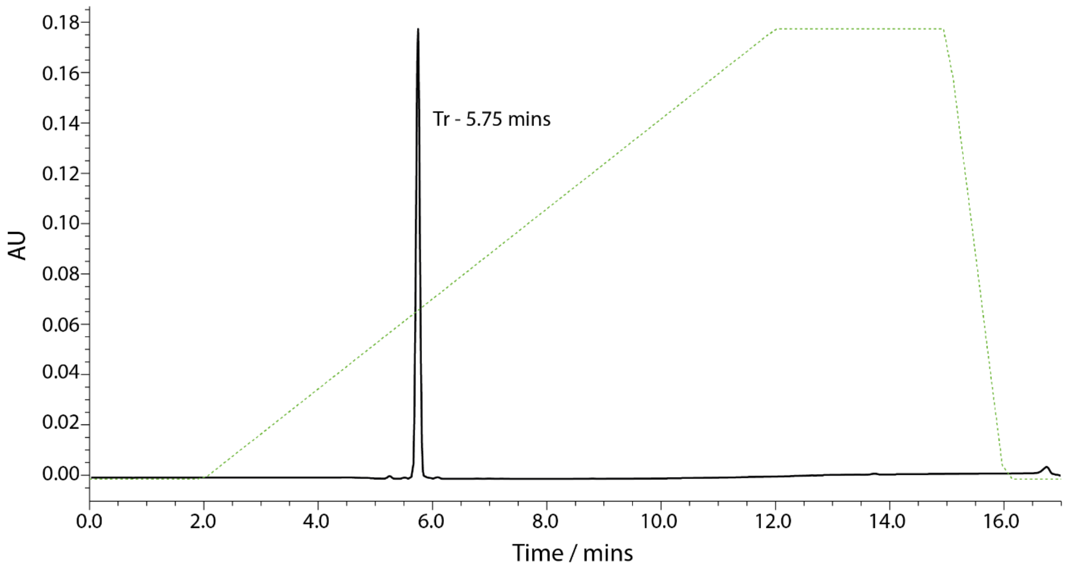

Analytical RP-HPLC trace of **Eu.Bn** [gradient: 0 − 100% acetonitrile in 0.05% v/v formic acid, over 10 minutes at 17 mL min^{-1}; T_R = 5.75 minutes].

4. Spectroscopic studies of Eu(III) Complexes

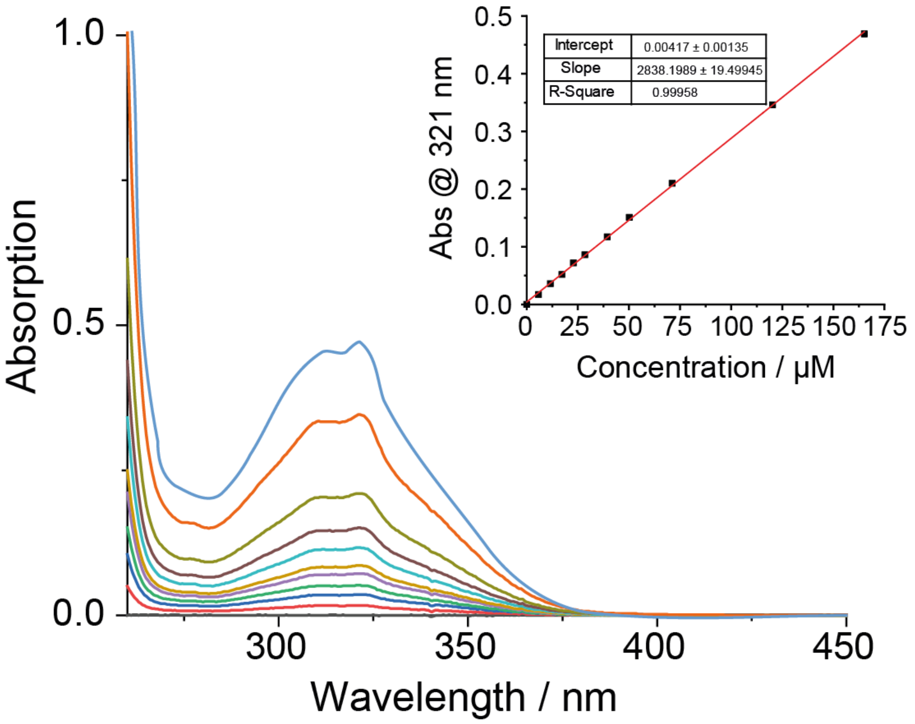

Figure S1: Change in absorption spectra of **Eu.1** as a function of concentration. Inset shows the fit to the experimental data, with a molar extinction coefficient, ε = 2,800 M^{-1} cm^{-1}. Conditions: 100 mM PBS buffer, pH 7.4.

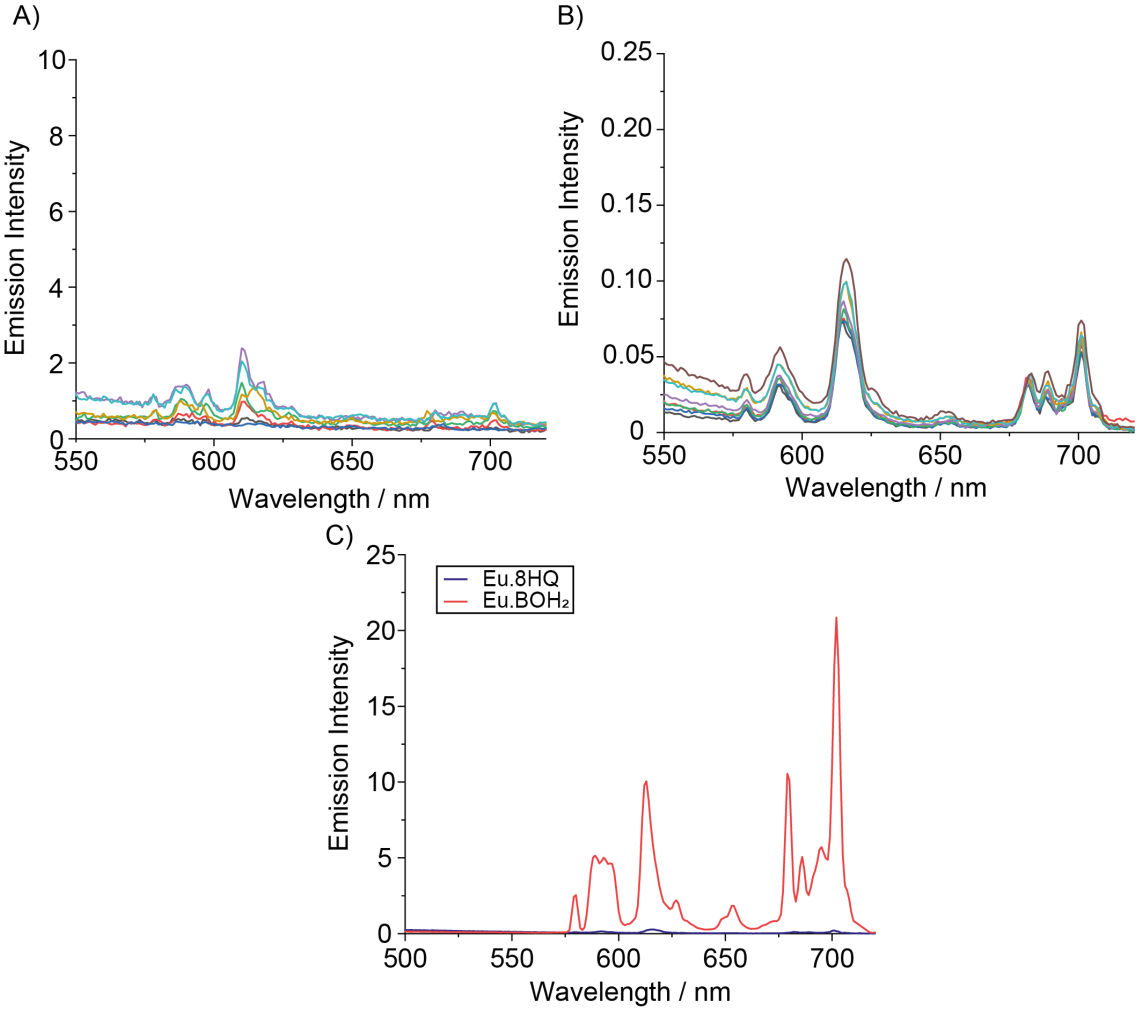

Figure S2: A) Emission spectra of **Eu.8HQ** (240 μM, 0.5 Abs) measured in different solvents, including CH_2Cl_2 (black), MeOH (red), diethyl ether (blue), acetonitrile (green), water (gold), and DMF (purple); B) Emission spectra of **Eu.8HQ** (100 μM) measured in water over the pH range 4.0–10.0; C) Comparison of emission spectra of **Eu.8HQ** and **Eu.1** in water (25 μM complex, pH 7.4, λ_{ex} = 321 nm).

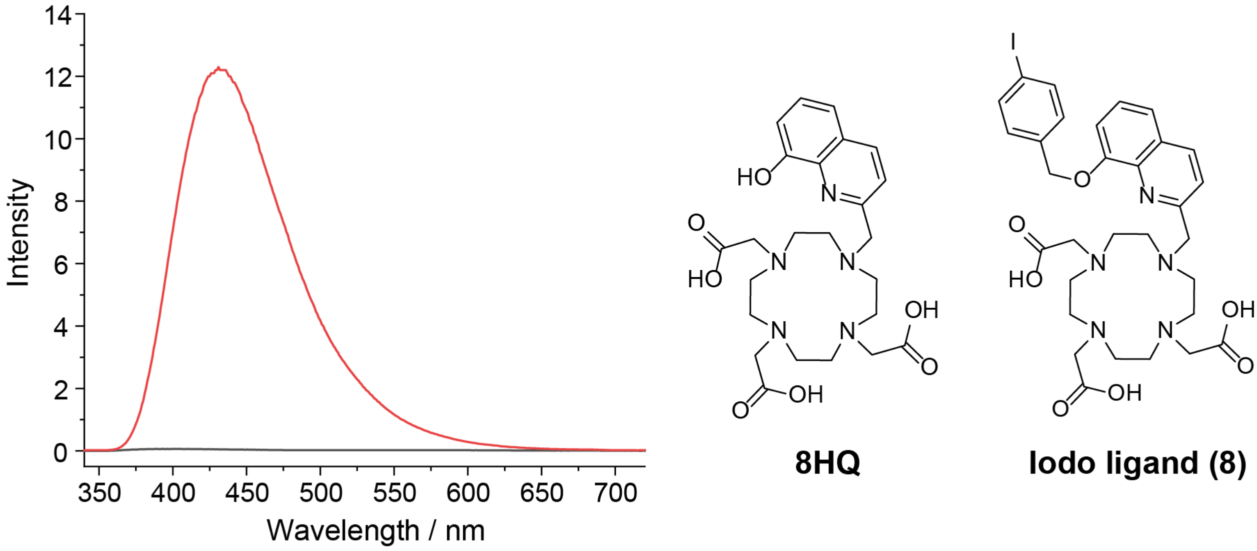

Figure S3: Emission spectra of ligand **8HQ** (black, λ_{exc} = 382 nm) compared with iodo ligand **8** (red λ_{exc} = 321 nm), measured in 100 mM PBS, pH 7.4, using samples at 0.5 absorbance.

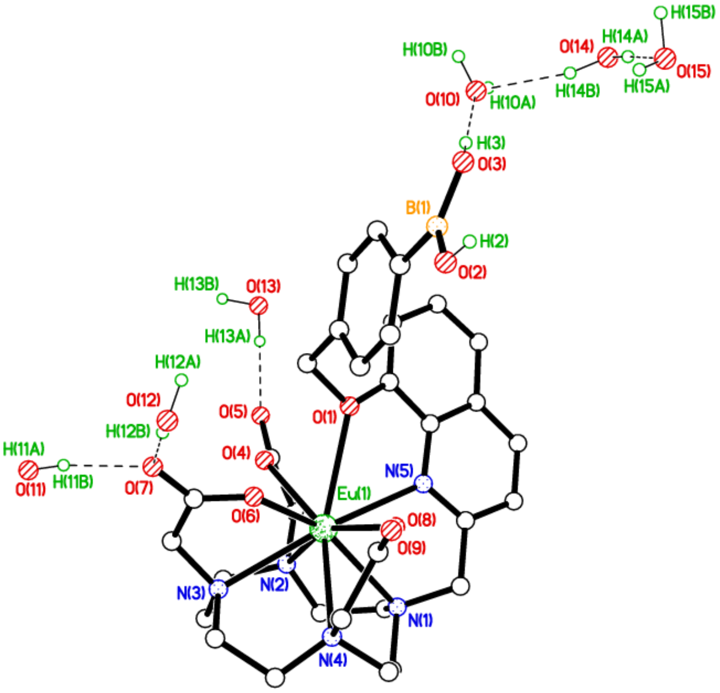

Figure S4. Calculated data for crystal structure of **Eu.1**. Crystals were prepared through slow evaporation of water over 72 hours. $C_{31}H_{37}BEuN_5O_9{\cdot}8H_2O$, the asymmetric unit, contains a 9-coordinate Eu(III) ion, binding only to the macrocyclic ligand. There are 8 water molecules of crystallisation per Eu(III) complex, and all water molecules of crystallisation are involved in H-bonding to each other or to oxygen atoms in the Eu(III) complex. The Eu(III) complexes are not directly H-bonded with each other. The nearest water molecule (O12) to europium is at a distance of 6.4 Å.

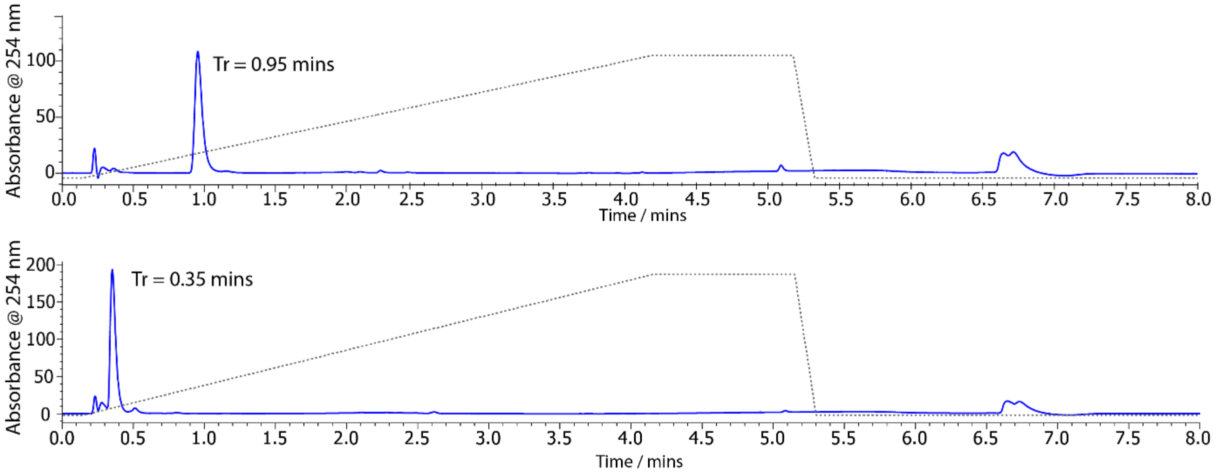

Figure S5: LC-MS data showing (upper) a single peak corresponding to **Eu.1** (250 μM in H_2O, pH 7.4); and (lower) a single peak for **Eu.8HQ**, following treatment of **Eu.1** with 250 μM $ONOO^-$ (H_2O, pH 7.4). Grey dashed line signifies percentage CH_3CN (5% - 95% in H_2O, over 4 minutes.

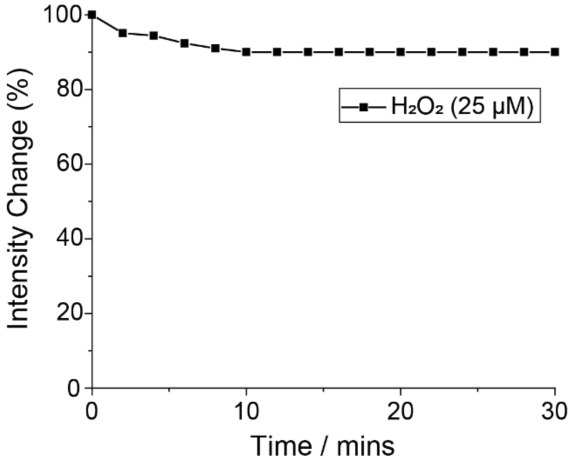

Figure S6: Minimal change in emission intensity of **Eu.1** (25 µM) following addition of 25 µM H_2O_2, measured in 100 mM PBS, pH 7.4.

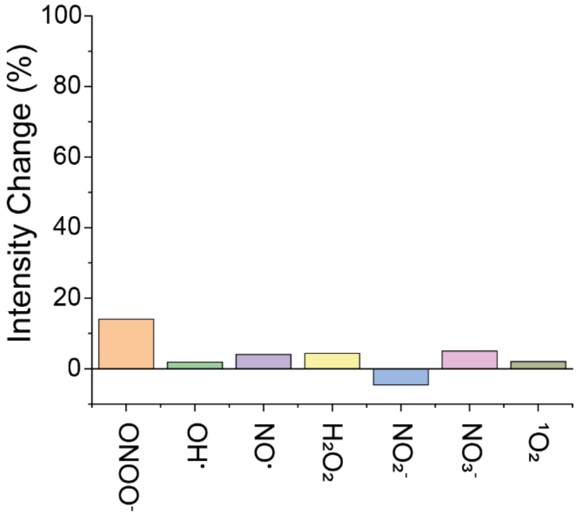

Figure S7: Selectivity studies for **Eu.Bn** (25 µM) showing minor intensity changes after incubation with a range of ROS/RNS over a 30 minute period. Measurements for $ONOO^-$ (100 µM), OH (1 mM), NO (sat), H_2O_2 (1 mM), NO_2^- (1 mM), NO_3^- (1 mM), and $^1O_2$ (sat) were carried out in 100 mM PBS, pH 7.4.

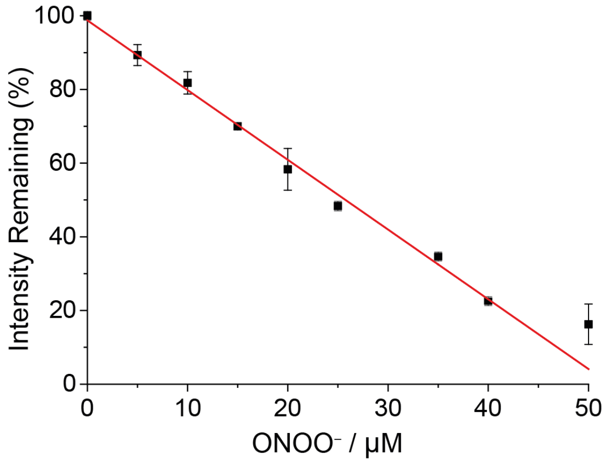

Figure S8: A) Standard curves for **Eu.1** (50 μM) time resolved (605 – 630 nm, 60 – 500 μs) emission changes in the presence of 0 – 50 μM $ONOO^-$ in human serum (λ_{ex} = 321 nm, λ_{em} = 605 – 630 nm, integration time = 60 – 400 μs).

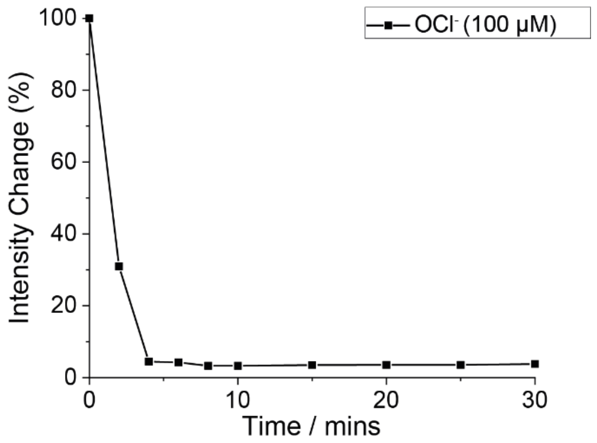

Figure S9: Emission intensity change of **Eu.1** (25 μM) in the presence of 100 μM OCl^- (PBS 100 mM, pH 7.4, λ_{exc} = 321 nm).

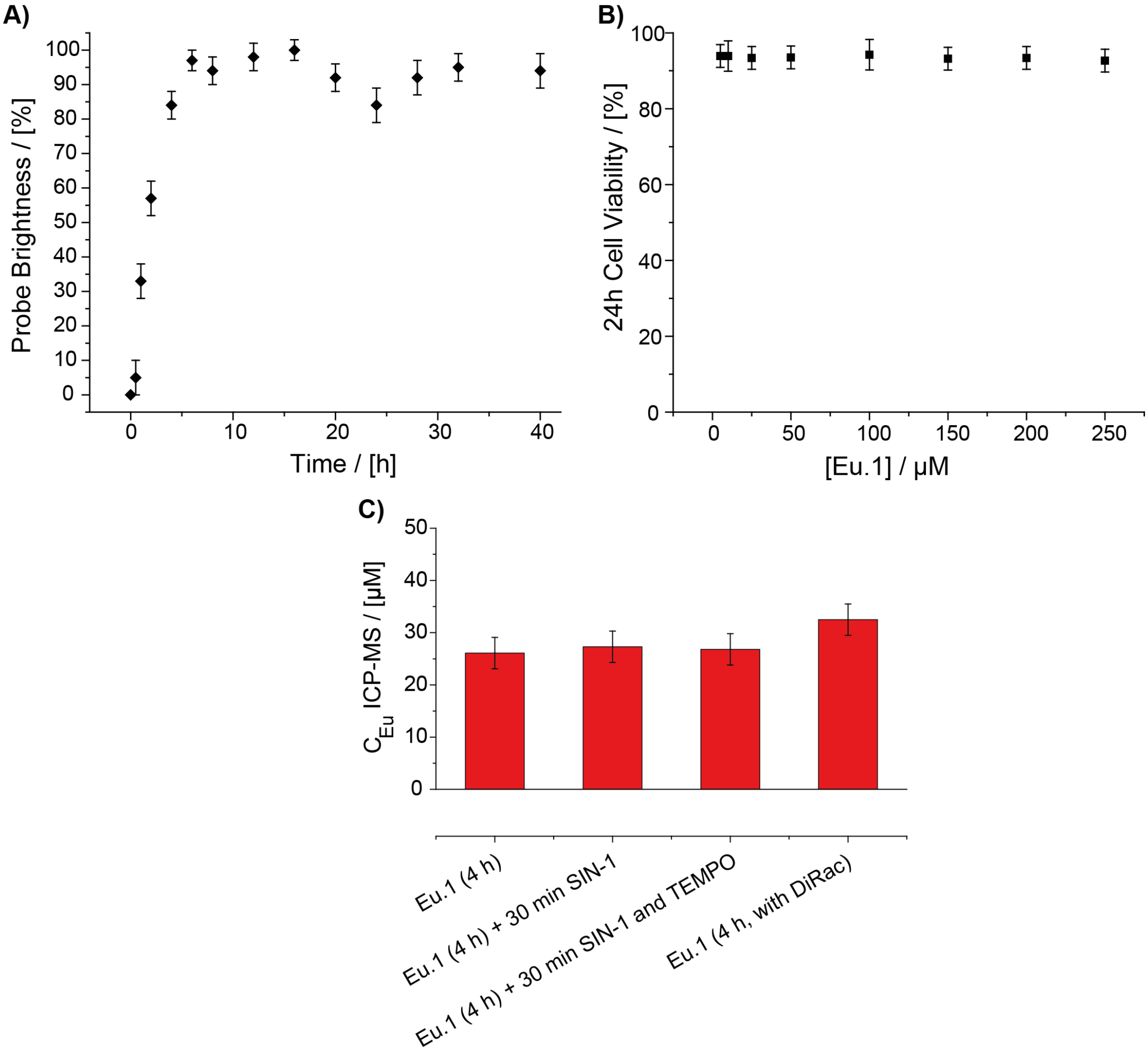

Figure S10: (A) Change in emission intensity of **Eu.1** in HeLa cells as a function of incubation time (maximum uptake at 4 hours). The off-trend data at approximately 25 hours corresponds to cell division occurring; B) Minimal variation of **Eu.1** intensity over a 24 hour time period; C) Concentration of Eu(III) in an average cell, determined by ICP-MS following incubation for 4 hours, with and without SIN-1 and TEMPO.

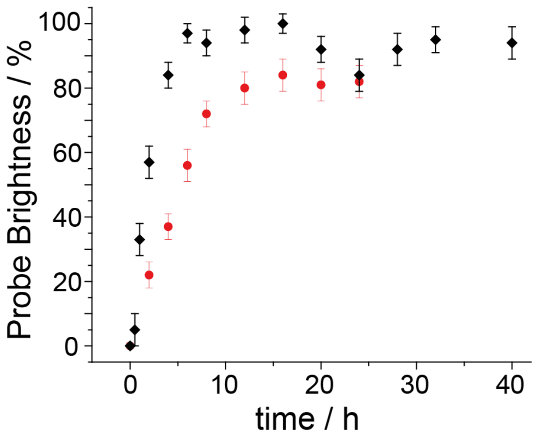

Figure S11: Comparison of the rate of uptake of **Eu.Bn** and **Eu.1** by HeLa cells, measured over a 40 hour time period. **Eu.Bn** exhibits a slower rate of uptake (maximum at 18 hours) compared with **Eu.1** (maximum at 4 hours).

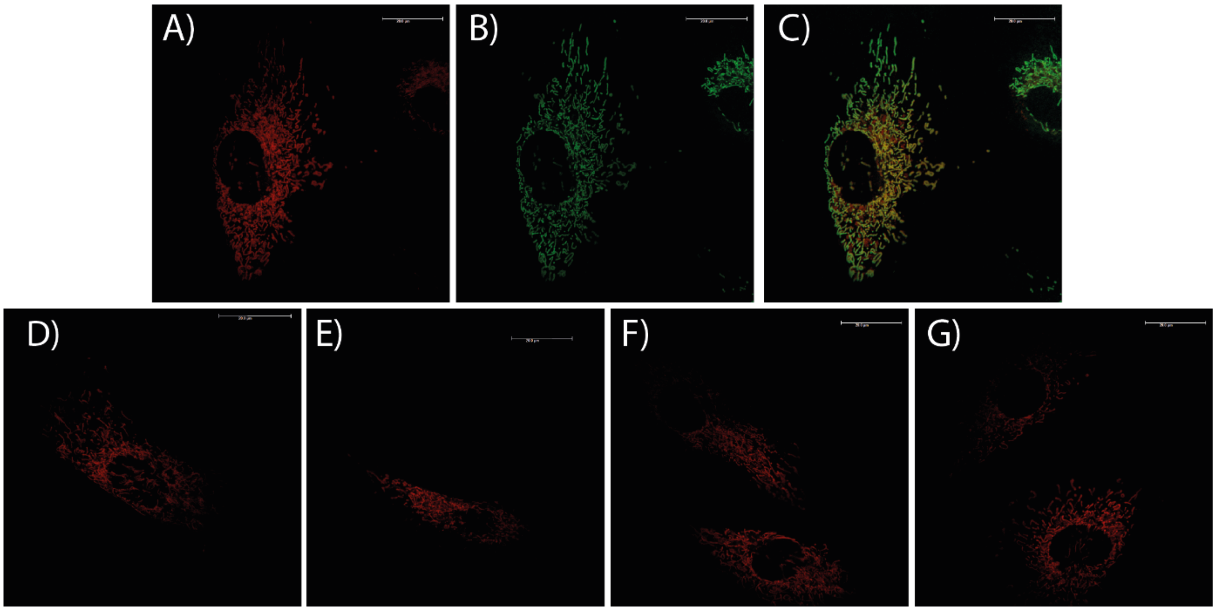

Figure S12: LSCM images showing localisation of control probe, **Eu.Bn,** in the mitochondria of HeLa cells. (A) **Eu.Bn** only (250 μM, 4h incubation); (B) MitoTracker Green™; (C) merge image (P=0.76). (D) LSCM image of **Eu.Bn** (250 μM, 4h); (E) **Eu.Bn** (250 μM, 4h) following 30 minute incubation with SIN-1 (500 μM); (F) **Eu.Bn** (250 μM, 4h) following 30 minute incubation with SIN-1 and TEMPO (500 μM and 1 mM respectively); and G) **Eu.Bn** (250 μM, 4h) following 30 minute incubation with TEMPO (1 mM).

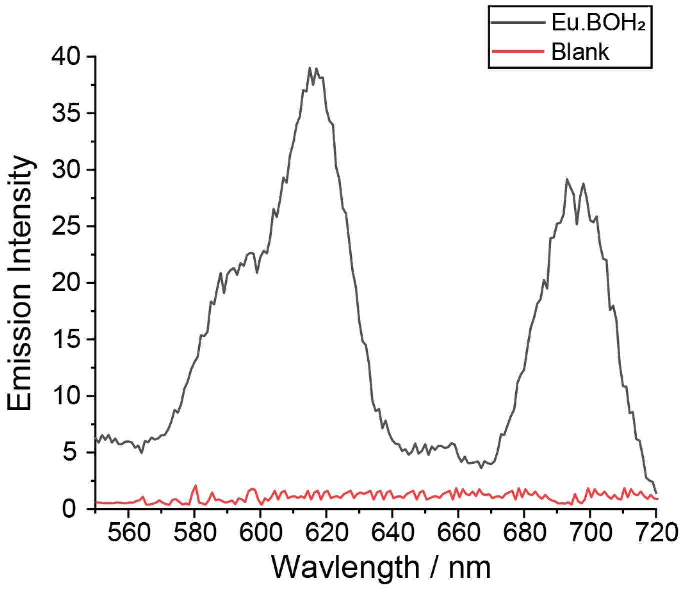

Figure S13: Time-resolved emission intensity of **Eu.1** following 24 hour incubation with THP cells (250 µM loading) measured in 5 mM PBS (pH 7.4) in a 96-flat glass bottom well plate, λ_{ex} = 321 nm, λ_{em} = 550 – 720 nm, integration time: 60 – 400 µs.

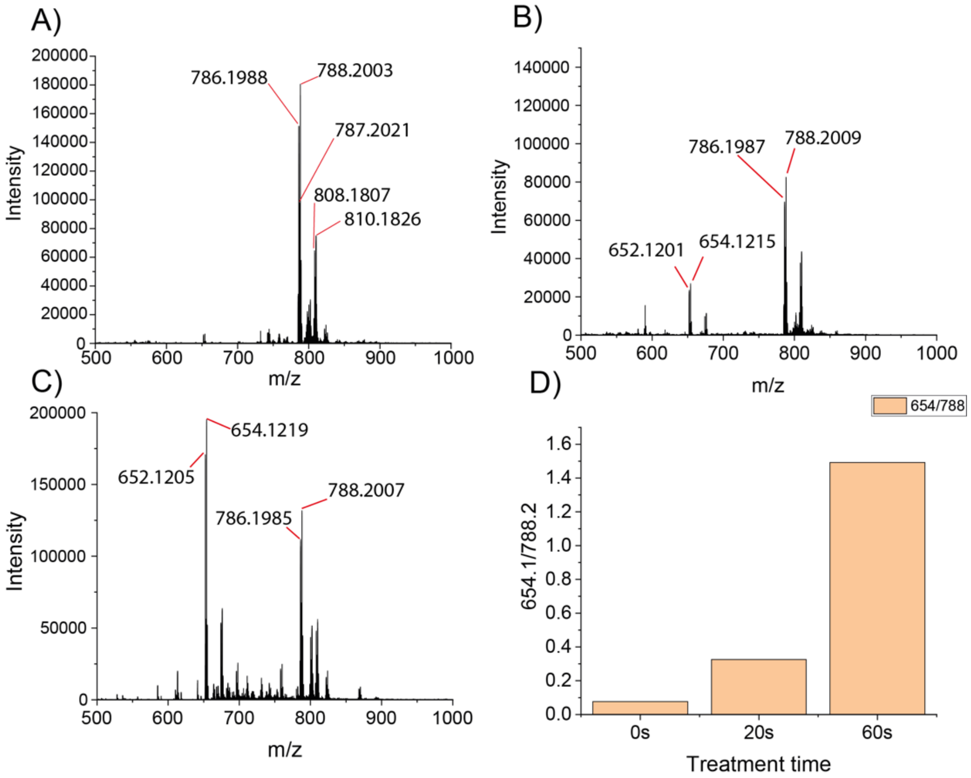

Figure S14: HR-TOF measurements of the reaction mixture containing **Eu.1** after plasma treatment using the kINPen MED device, following A) 0 seconds, B) 20 seconds, and C) 60 seconds treatment time. Signal corresponding to the formation of the product, **Eu.8HQ** (m/z = 654.122) increases, whilst signal for **Eu.1** (m/z = 788.171) decreases, with increasing treatment time.

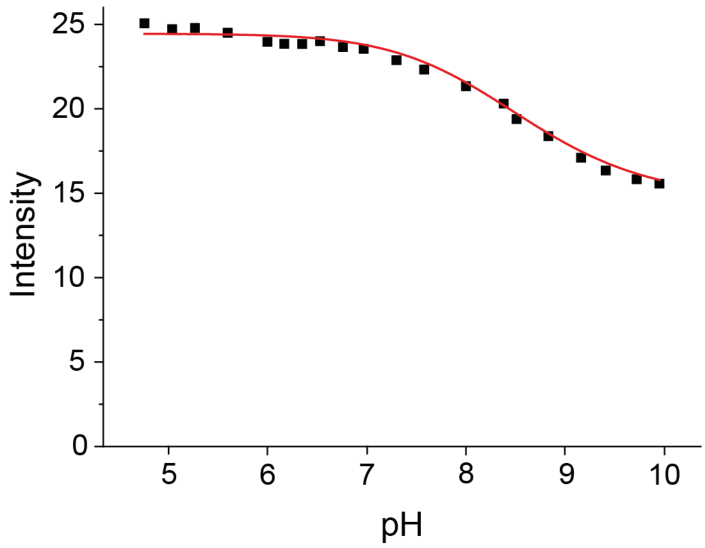

Figure S15: pH profile of **Eu.1**

X-ray Crystallography

Diffraction data were collected on Station 11.3.1 at the ALS synchrotron using a Bruker D8 diffractometer equipped with a Photon 100 detector.[5] Further details are given in the tables below and in the cif (CCDC 1965719 contains the supplementary crystallographic data for this paper. These data can be obtained free of charge from The Cambridge Crystallographic Data Centre via www.ccdc.cam.ac.uk/structures). The data were corrected for polarisation and Lp effects and for absorption.[6] The structure was solved via a charge flipping dual space algorithm[7] and refined by full-matrix least-squares on F^2.[8] Six of the 8 unique water molecules were refined as point atoms. H atom positions on these were located via difference maps and refined with O–H and H···H distance restraints. Due to the nature of the packing it is likely the water molecules are somewhat disordered in terms of H atom positions. There are 8 water molecules of crystallisation per Eu complex. Two of these were substantially disordered and were refined as diffuse areas of electron density by the Platon Squeeze procedure.[9] Platon Squeeze recovered 106 electrons per unit cell, equally distributed between two voids. This equates to 8 extra water molecules per unit cell and agrees with the point atom observations. They are in the area of a 2-fold disordered carboxylate group C(31)/O(8)/O(9), which has major component occupancy of 55.2(4)%. All water molecules of crystallisation are involved in H-bonding to each other or to O atoms in the Eu complex. They therefore provide H-bonded bridging links between Eu complexes. The Eu complexes are not directly H-bonded with each other.

Crystallographic Data Tables for Eu.1.

Crystal data

$C_{31}H_{37}BEuN_5O_9 \cdot 8(H_2O)$	$F(000) = 1912$
$M_r = 930.55$	$D_x = 1.610$ Mg m^{-3}
Monoclinic, $P2_1/n$	Synchrotron radiation, λ = 0.7288 Å
a = 13.2696 (5) Å	Cell parameters from 9168 reflections
b = 15.8873 (6) Å	θ = 3.1–36.0°
c = 18.2435 (7) Å	μ = 1.82 mm^{-1}
β = 93.300 (2)°	T = 100 K
V = 3839.7 (3) Å^3	Tablet, colourless
Z = 4	0.20 × 0.19 × 0.09 mm^3

Data collection

Bruker D8 with PHOTON 100 detector diffractometer	16877 independent reflections
Radiation source: Advanced Light Source station 11.3.1	15100 reflections with $I > 2\sigma(I)$
Silicon 111 monochromator	$R_{int} = 0.063$
Detector resolution: 10.42 pixels mm^{-1}	$\theta_{max} = 36.0°$, $\theta_{min} = 1.7°$
ω/θ shutterless scans	$h = -21 \rightarrow 21$
Absorption correction: multi-scan *SADABS* v2016/2, Sheldrick, G.M., (2012)	$k = -25 \rightarrow 25$
$T_{min} = 0.713$, $T_{max} = 0.854$	$l = -29 \rightarrow 29$
159626 measured reflections	

Refinement

Refinement on F^2	Secondary atom site location: difference Fourier map
Least-squares matrix: full	Hydrogen site location: mixed
$R[F^2 > 2\sigma(F^2)] = 0.027$	H atoms treated by a mixture of independent and constrained refinement
$wR(F^2) = 0.069$	$w = 1/[\sigma^2(F_o^2) + (0.0296P)^2 + 2.9825P]$ where $P = (F_o^2 + 2F_c^2)/3$
$S = 1.03$	$(\Delta/\sigma)_{max} = 0.002$
16877 reflections	$\Delta\rangle_{max} = 0.89$ e Å^{-3}
540 parameters	$\Delta\rangle_{min} = -1.03$ e Å^{-3}
96 restraints	Extinction correction: *SHELXL2018*/3 (Sheldrick 2018), $Fc^* = kFc[1+0.001xFc^2\lambda^3/\sin(2\theta)]^{-1/4}$
Primary atom site location: iterative	Extinction coefficient: 0.00220 (13)

Fractional atomic coordinates and isotropic or equivalent isotropic displacement parameters (Å^2).

	x	*y*	*z*	U_{iso}*/U_{eq}	Occ. (<1)
Eu1	0.18574 (2)	0.44845 (2)	0.31416 (2)	0.00983 (2)	
N1	0.00531 (9)	0.48358 (8)	0.24942 (6)	0.0143 (2)	
C1	-0.07868 (11)	0.46342 (10)	0.29731 (8)	0.0153 (2)	
H1A	-0.143479	0.464797	0.267596	0.018*	
H1B	-0.081544	0.506854	0.336049	0.018*	
C2	-0.06553 (11)	0.37772 (10)	0.33285 (8)	0.0159 (2)	
H2A	-0.120597	0.368143	0.366268	0.019*	
H2B	-0.070294	0.333693	0.294399	0.019*	
N2	0.03299 (9)	0.37061 (7)	0.37481 (6)	0.01222 (19)	
C3	0.05786 (11)	0.28061 (9)	0.38858 (8)	0.0148 (2)	
H3A	-0.004258	0.249890	0.399906	0.018*	
H3B	0.106211	0.276211	0.431809	0.018*	
C4	0.10326 (11)	0.24025 (9)	0.32308 (8)	0.0149 (2)	
H4A	0.118683	0.180528	0.334431	0.018*	
H4B	0.053269	0.241708	0.280674	0.018*	
N3	0.19696 (9)	0.28339 (8)	0.30289 (6)	0.01282 (19)	
C5	0.22125 (11)	0.25590 (9)	0.22783 (7)	0.0148 (2)	
H5A	0.209572	0.194558	0.222948	0.018*	
H5B	0.293518	0.266781	0.220806	0.018*	
C6	0.15768 (12)	0.30148 (9)	0.16907 (8)	0.0162 (2)	
H6A	0.178298	0.283774	0.120101	0.019*	
H6B	0.085888	0.286032	0.172839	0.019*	
N4	0.16902 (10)	0.39401 (8)	0.17650 (7)	0.0160 (2)	
C7	0.08335 (14)	0.43673 (10)	0.13538 (8)	0.0215 (3)	
H7A	0.068574	0.407336	0.088132	0.026*	
H7B	0.102755	0.495361	0.124406	0.026*	
C8	-0.01060 (13)	0.43737 (10)	0.17881 (8)	0.0193 (3)	
H8A	-0.066499	0.464106	0.148989	0.023*	
H8B	-0.030546	0.378670	0.188988	0.023*	
C9	0.00071 (12)	0.57538 (10)	0.23436 (8)	0.0169 (2)	
H9A	-0.069450	0.591248	0.218845	0.020*	
H9B	0.044119	0.588792	0.193577	0.020*	
N5	0.11551 (9)	0.59798 (7)	0.33996 (6)	0.01254 (19)	
C10	0.03491 (11)	0.62569 (9)	0.30089 (8)	0.0148 (2)	
C11	-0.01626 (12)	0.70049 (10)	0.31816 (9)	0.0199 (3)	
H11	-0.072736	0.718897	0.287994	0.024*	
C12	0.01597 (13)	0.74620 (10)	0.37851 (9)	0.0202 (3)	

H12	-0.020263	0.794737	0.392238	0.024*	
C13	0.10360 (12)	0.72069 (9)	0.42031 (8)	0.0157 (2)	
C14	0.14350 (13)	0.76577 (9)	0.48236 (8)	0.0188 (3)	
H14	0.110279	0.814785	0.498496	0.023*	
C15	0.23030 (13)	0.73821 (9)	0.51893 (8)	0.0188 (3)	
H15	0.255042	0.766865	0.561991	0.023*	
C16	0.28385 (12)	0.66783 (9)	0.49379 (8)	0.0165 (2)	
H16	0.345118	0.650871	0.519024	0.020*	
C17	0.24734 (11)	0.62382 (8)	0.43279 (7)	0.0130 (2)	
C18	0.15325 (10)	0.64684 (8)	0.39719 (7)	0.0125 (2)	
O1	0.29577 (8)	0.55927 (6)	0.39975 (6)	0.01358 (18)	
C19	0.39585 (11)	0.53670 (9)	0.43064 (8)	0.0148 (2)	
H19A	0.411904	0.478293	0.416419	0.018*	
H19B	0.396731	0.539294	0.484887	0.018*	
C20	0.47436 (10)	0.59580 (9)	0.40345 (8)	0.0135 (2)	
C21	0.49094 (11)	0.59670 (10)	0.32845 (8)	0.0167 (2)	
H21	0.451066	0.562334	0.295511	0.020*	
C22	0.56599 (11)	0.64803 (10)	0.30201 (8)	0.0179 (3)	
H22	0.577124	0.647432	0.251034	0.021*	
C23	0.62546 (11)	0.70051 (10)	0.34874 (9)	0.0175 (3)	
C24	0.60755 (12)	0.69883 (10)	0.42365 (9)	0.0178 (3)	
H24	0.646695	0.733677	0.456645	0.021*	
C25	0.53326 (11)	0.64693 (9)	0.45086 (8)	0.0164 (2)	
H25	0.522874	0.646530	0.501946	0.020*	
B1	0.70877 (14)	0.75894 (12)	0.31732 (11)	0.0220 (3)	
O2	0.73645 (9)	0.74287 (9)	0.24772 (7)	0.0262 (3)	
H2	0.7769 (17)	0.7764 (14)	0.2307 (14)	0.039*	
O3	0.75143 (12)	0.82432 (9)	0.35454 (10)	0.0361 (3)	
H3	0.730 (2)	0.8423 (19)	0.3930 (11)	0.054*	
C26	0.02875 (10)	0.41369 (9)	0.44602 (7)	0.0135 (2)	
H26A	-0.014445	0.381161	0.478252	0.016*	
H26B	-0.001677	0.470149	0.438459	0.016*	
C27	0.13425 (11)	0.42260 (9)	0.48296 (7)	0.0129 (2)	
O4	0.20747 (8)	0.41701 (7)	0.44080 (6)	0.01418 (18)	
O5	0.14136 (9)	0.43650 (8)	0.55005 (6)	0.0190 (2)	
C28	0.28353 (11)	0.26048 (9)	0.35337 (8)	0.0143 (2)	
H28A	0.304470	0.201931	0.343595	0.017*	
H28B	0.263420	0.263602	0.404726	0.017*	
C29	0.37168 (11)	0.32003 (9)	0.34311 (7)	0.0132 (2)	
O6	0.35056 (8)	0.39161 (6)	0.31476 (6)	0.01443 (18)	

O7	0.45825 (8)	0.29622 (7)	0.36377 (6)	0.0191 (2)	
C30	0.26423 (14)	0.42094 (10)	0.14483 (9)	0.0212 (3)	
H30A	0.251399	0.429859	0.091397	0.025*	0.552 (4)
H30B	0.314518	0.375159	0.151576	0.025*	0.552 (4)
H30C	0.260990	0.410792	0.091202	0.025*	0.448 (4)
H30D	0.322168	0.389228	0.167546	0.025*	0.448 (4)
C31	0.3098 (3)	0.50345 (19)	0.17989 (18)	0.0152 (5)	0.552 (4)
O8	0.25618 (10)	0.53696 (7)	0.22764 (6)	0.0190 (2)	
O9	0.38913 (18)	0.53287 (14)	0.15607 (13)	0.0231 (6)	0.552 (4)
C31X	0.2757 (3)	0.5138 (2)	0.1609 (2)	0.0162 (7)	0.448 (4)
O9X	0.3179 (2)	0.55857 (17)	0.11610 (17)	0.0263 (8)	0.448 (4)
O10	0.69449 (13)	0.89458 (9)	0.48375 (10)	0.0362 (3)	
H10B	0.7522 (12)	0.893 (2)	0.5038 (15)	0.054*	
H10A	0.6535 (16)	0.916 (2)	0.5092 (14)	0.054*	
O11	0.47516 (13)	0.14363 (9)	0.44118 (8)	0.0336 (3)	
H11A	0.4290 (17)	0.1389 (17)	0.4690 (14)	0.050*	
H11B	0.476 (2)	0.1901 (12)	0.4212 (14)	0.050*	
O12	0.63237 (11)	0.35490 (11)	0.30445 (11)	0.0447 (5)	
H12A	0.656 (2)	0.3936 (16)	0.3320 (16)	0.067*	
H12B	0.5807 (17)	0.3385 (19)	0.3254 (16)	0.067*	
O13	0.30617 (9)	0.51184 (8)	0.61379 (7)	0.0228 (2)	
H13B	0.3406 (16)	0.4709 (12)	0.6313 (13)	0.034*	
H13A	0.2555 (13)	0.4885 (14)	0.5916 (13)	0.034*	
O14	0.65733 (13)	1.06341 (9)	0.45719 (8)	0.0311 (3)	
H14A	0.688 (2)	1.0779 (16)	0.4214 (12)	0.047*	
H14B	0.661 (2)	1.0117 (9)	0.4616 (15)	0.047*	
O15	0.76350 (19)	1.09554 (16)	0.33834 (14)	0.0703 (7)	
H15B	0.815 (2)	1.115 (3)	0.366 (2)	0.105*	
H15A	0.798 (3)	1.055 (2)	0.318 (2)	0.105*	

Geometric parameters (Å, º)

Eu1—O8	2.3478 (11)	C15—H15	0.9500
Eu1—O4	2.3650 (10)	C16—C17	1.378 (2)
Eu1—O6	2.3657 (10)	C16—H16	0.9500
Eu1—N5	2.6046 (12)	C17—O1	1.3685 (17)
Eu1—N3	2.6354 (12)	C17—C18	1.422 (2)
Eu1—N4	2.6534 (12)	O1—C19	1.4572 (18)
Eu1—N1	2.6671 (12)	C19—C20	1.508 (2)
Eu1—N2	2.6684 (12)	C19—H19A	0.9900
Eu1—O1	2.7216 (10)	C19—H19B	0.9900

N1—C9	1.4846 (19)	C20—C25	1.393 (2)
N1—C8	1.4872 (19)	C20—C21	1.398 (2)
N1—C1	1.4901 (19)	C21—C22	1.395 (2)
C1—C2	1.514 (2)	C21—H21	0.9500
C1—H1A	0.9900	C22—C23	1.403 (2)
C1—H1B	0.9900	C22—H22	0.9500
C2—N2	1.4807 (19)	C23—C24	1.401 (2)
C2—H2A	0.9900	C23—B1	1.576 (2)
C2—H2B	0.9900	C24—C25	1.398 (2)
N2—C26	1.4725 (18)	C24—H24	0.9500
N2—C3	1.4855 (18)	C25—H25	0.9500
C3—C4	1.512 (2)	B1—O3	1.348 (3)
C3—H3A	0.9900	B1—O2	1.366 (2)
C3—H3B	0.9900	O2—H2	0.829 (13)
C4—N3	1.4846 (18)	O3—H3	0.822 (14)
C4—H4A	0.9900	C26—C27	1.525 (2)
C4—H4B	0.9900	C26—H26A	0.9900
N3—C28	1.4760 (19)	C26—H26B	0.9900
N3—C5	1.4902 (18)	C27—O5	1.2419 (17)
C5—C6	1.510 (2)	C27—O4	1.2766 (17)
C5—H5A	0.9900	C28—C29	1.524 (2)
C5—H5B	0.9900	C28—H28A	0.9900
C6—N4	1.4832 (19)	C28—H28B	0.9900
C6—H6A	0.9900	C29—O7	1.2469 (18)
C6—H6B	0.9900	C29—O6	1.2738 (17)
N4—C30	1.482 (2)	C30—C31X	1.511 (4)
N4—C7	1.489 (2)	C30—C31	1.565 (3)
C7—C8	1.515 (2)	C30—H30A	0.9900
C7—H7A	0.9900	C30—H30B	0.9900
C7—H7B	0.9900	C30—H30C	0.9900
C8—H8A	0.9900	C30—H30D	0.9900
C8—H8B	0.9900	C31—O9	1.253 (4)
C9—C10	1.502 (2)	C31—O8	1.272 (3)
C9—H9A	0.9900	O8—C31X	1.312 (4)
C9—H9B	0.9900	C31X—O9X	1.240 (4)
N5—C10	1.3258 (18)	O10—H10B	0.829 (13)
N5—C18	1.3723 (18)	O10—H10A	0.808 (13)
C10—C11	1.413 (2)	O11—H11A	0.821 (13)
C11—C12	1.367 (2)	O11—H11B	0.823 (13)
C11—H11	0.9500	O12—H12A	0.845 (14)

C12—C13	1.413 (2)	O12—H12B	0.845 (14)
C12—H12	0.9500	O13—H13B	0.847 (13)
C13—C14	1.416 (2)	O13—H13A	0.851 (13)
C13—C18	1.4213 (19)	O14—H14A	0.819 (13)
C14—C15	1.369 (2)	O14—H14B	0.826 (13)
C14—H14	0.9500	O15—H15B	0.877 (14)
C15—C16	1.415 (2)	O15—H15A	0.881 (14)
O8—Eu1—O4	138.79 (4)	C10—N5—C18	117.96 (12)
O8—Eu1—O6	80.09 (4)	C10—N5—Eu1	119.45 (9)
O4—Eu1—O6	81.62 (4)	C18—N5—Eu1	122.33 (9)
O8—Eu1—N5	74.67 (4)	N5—C10—C11	122.85 (14)
O4—Eu1—N5	92.34 (4)	N5—C10—C9	117.05 (12)
O6—Eu1—N5	133.45 (4)	C11—C10—C9	120.07 (13)
O8—Eu1—N3	121.13 (4)	C12—C11—C10	119.62 (14)
O4—Eu1—N3	82.06 (4)	C12—C11—H11	120.2
O6—Eu1—N3	64.16 (4)	C10—C11—H11	120.2
N5—Eu1—N3	160.75 (4)	C11—C12—C13	119.47 (14)
O8—Eu1—N4	64.77 (4)	C11—C12—H12	120.3
O4—Eu1—N4	148.71 (4)	C13—C12—H12	120.3
O6—Eu1—N4	84.65 (4)	C12—C13—C14	123.00 (13)
N5—Eu1—N4	117.20 (4)	C12—C13—C18	117.25 (13)
N3—Eu1—N4	66.66 (4)	C14—C13—C18	119.75 (14)
O8—Eu1—N1	87.65 (4)	C15—C14—C13	119.54 (14)
O4—Eu1—N1	122.19 (4)	C15—C14—H14	120.2
O6—Eu1—N1	152.63 (4)	C13—C14—H14	120.2
N5—Eu1—N1	64.39 (4)	C14—C15—C16	121.24 (14)
N3—Eu1—N1	103.12 (4)	C14—C15—H15	119.4
N4—Eu1—N1	67.98 (4)	C16—C15—H15	119.4
O8—Eu1—N2	153.85 (4)	C17—C16—C15	120.18 (15)
O4—Eu1—N2	63.25 (4)	C17—C16—H16	119.9
O6—Eu1—N2	123.04 (4)	C15—C16—H16	119.9
N5—Eu1—N2	93.41 (4)	O1—C17—C16	125.71 (13)
N3—Eu1—N2	67.59 (4)	O1—C17—C18	114.45 (12)
N4—Eu1—N2	102.54 (4)	C16—C17—C18	119.80 (13)
N1—Eu1—N2	66.21 (4)	N5—C18—C13	122.52 (13)
O8—Eu1—O1	77.12 (4)	N5—C18—C17	118.32 (12)
O4—Eu1—O1	62.94 (3)	C13—C18—C17	119.12 (13)
O6—Eu1—O1	77.23 (3)	C17—O1—C19	117.05 (11)
N5—Eu1—O1	59.47 (3)	C17—O1—Eu1	119.17 (8)

N3—Eu1—O1	131.12 (3)	C19—O1—Eu1	120.63 (8)
N4—Eu1—O1	140.06 (4)	O1—C19—C20	110.56 (11)
N1—Eu1—O1	123.85 (3)	O1—C19—H19A	109.5
N2—Eu1—O1	117.21 (3)	C20—C19—H19A	109.5
C9—N1—C8	108.81 (11)	O1—C19—H19B	109.5
C9—N1—C1	107.19 (11)	C20—C19—H19B	109.5
C8—N1—C1	109.16 (12)	H19A—C19—H19B	108.1
C9—N1—Eu1	108.29 (9)	C25—C20—C21	119.17 (13)
C8—N1—Eu1	111.06 (9)	C25—C20—C19	122.17 (13)
C1—N1—Eu1	112.21 (8)	C21—C20—C19	118.61 (13)
N1—C1—C2	111.89 (12)	C22—C21—C20	120.01 (14)
N1—C1—H1A	109.2	C22—C21—H21	120.0
C2—C1—H1A	109.2	C20—C21—H21	120.0
N1—C1—H1B	109.2	C21—C22—C23	121.69 (14)
C2—C1—H1B	109.2	C21—C22—H22	119.2
H1A—C1—H1B	107.9	C23—C22—H22	119.2
N2—C2—C1	111.50 (12)	C24—C23—C22	117.42 (14)
N2—C2—H2A	109.3	C24—C23—B1	121.86 (15)
C1—C2—H2A	109.3	C22—C23—B1	120.72 (14)
N2—C2—H2B	109.3	C25—C24—C23	121.35 (14)
C1—C2—H2B	109.3	C25—C24—H24	119.3
H2A—C2—H2B	108.0	C23—C24—H24	119.3
C26—N2—C2	110.07 (11)	C20—C25—C24	120.35 (13)
C26—N2—C3	108.52 (11)	C20—C25—H25	119.8
C2—N2—C3	109.99 (11)	C24—C25—H25	119.8
C26—N2—Eu1	102.64 (8)	O3—B1—O2	118.99 (16)
C2—N2—Eu1	114.80 (8)	O3—B1—C23	123.62 (16)
C3—N2—Eu1	110.46 (8)	O2—B1—C23	117.36 (17)
N2—C3—C4	111.68 (11)	B1—O2—H2	116.2 (19)
N2—C3—H3A	109.3	B1—O3—H3	123 (2)
C4—C3—H3A	109.3	N2—C26—C27	110.63 (11)
N2—C3—H3B	109.3	N2—C26—H26A	109.5
C4—C3—H3B	109.3	C27—C26—H26A	109.5
H3A—C3—H3B	107.9	N2—C26—H26B	109.5
N3—C4—C3	112.20 (11)	C27—C26—H26B	109.5
N3—C4—H4A	109.2	H26A—C26—H26B	108.1
C3—C4—H4A	109.2	O5—C27—O4	126.08 (14)
N3—C4—H4B	109.2	O5—C27—C26	117.88 (13)
C3—C4—H4B	109.2	O4—C27—C26	116.01 (12)
H4A—C4—H4B	107.9	O5—C27—Eu1	155.77 (11)

C28—N3—C4	111.36 (11)	C26—C27—Eu1	80.16 (7)
C28—N3—C5	107.42 (11)	C27—O4—Eu1	121.31 (9)
C4—N3—C5	108.74 (11)	N3—C28—C29	110.22 (11)
C28—N3—Eu1	104.01 (8)	N3—C28—H28A	109.6
C4—N3—Eu1	112.84 (8)	C29—C28—H28A	109.6
C5—N3—Eu1	112.30 (8)	N3—C28—H28B	109.6
N3—C5—C6	111.73 (11)	C29—C28—H28B	109.6
N3—C5—H5A	109.3	H28A—C28—H28B	108.1
C6—C5—H5A	109.3	O7—C29—O6	124.81 (13)
N3—C5—H5B	109.3	O7—C29—C28	118.34 (13)
C6—C5—H5B	109.3	O6—C29—C28	116.84 (12)
H5A—C5—H5B	107.9	O7—C29—Eu1	157.69 (10)
N4—C6—C5	111.15 (12)	C28—C29—Eu1	80.05 (8)
N4—C6—H6A	109.4	C29—O6—Eu1	121.63 (9)
C5—C6—H6A	109.4	N4—C30—C31X	106.58 (18)
N4—C6—H6B	109.4	N4—C30—C31	113.59 (15)
C5—C6—H6B	109.4	N4—C30—H30A	108.8
H6A—C6—H6B	108.0	C31—C30—H30A	108.8
C30—N4—C6	109.55 (12)	N4—C30—H30B	108.8
C30—N4—C7	108.37 (12)	C31—C30—H30B	108.8
C6—N4—C7	109.59 (12)	H30A—C30—H30B	107.7
C30—N4—Eu1	104.37 (9)	N4—C30—H30C	110.4
C6—N4—Eu1	114.34 (8)	C31X—C30—H30C	110.4
C7—N4—Eu1	110.37 (9)	N4—C30—H30D	110.4
N4—C7—C8	111.53 (12)	C31X—C30—H30D	110.4
N4—C7—H7A	109.3	H30C—C30—H30D	108.6
C8—C7—H7A	109.3	O9—C31—O8	126.5 (3)
N4—C7—H7B	109.3	O9—C31—C30	118.9 (2)
C8—C7—H7B	109.3	O8—C31—C30	114.4 (2)
H7A—C7—H7B	108.0	O9—C31—Eu1	149.3 (3)
N1—C8—C7	112.10 (14)	C30—C31—Eu1	83.04 (12)
N1—C8—H8A	109.2	C31—O8—Eu1	118.00 (15)
C7—C8—H8A	109.2	C31X—O8—Eu1	124.34 (18)
N1—C8—H8B	109.2	O9X—C31X—O8	124.9 (3)
C7—C8—H8B	109.2	O9X—C31X—C30	118.5 (3)
H8A—C8—H8B	107.9	O8—C31X—C30	115.5 (3)
N1—C9—C10	111.47 (11)	H10B—O10—H10A	113 (2)
N1—C9—H9A	109.3	H11A—O11—H11B	112 (2)
C10—C9—H9A	109.3	H12A—O12—H12B	104 (2)
N1—C9—H9B	109.3	H13B—O13—H13A	103.8 (19)

C10—C9—H9B	109.3	H14A—O14—H14B	109 (2)
H9A—C9—H9B	108.0	H15B—O15—H15A	95 (2)
C9—N1—C1—C2	-164.72 (12)	C16—C17—C18—N5	-175.28 (12)
C8—N1—C1—C2	77.59 (14)	O1—C17—C18—C13	-170.92 (12)
Eu1—N1—C1—C2	-45.96 (13)	C16—C17—C18—C13	6.81 (19)
N1—C1—C2—N2	55.82 (15)	C16—C17—O1—C19	-1.78 (19)
C1—C2—N2—C26	78.42 (14)	C18—C17—O1—C19	175.80 (11)
C1—C2—N2—C3	-162.06 (11)	C16—C17—O1—Eu1	158.31 (11)
C1—C2—N2—Eu1	-36.77 (14)	C18—C17—O1—Eu1	-24.11 (14)
C26—N2—C3—C4	-157.60 (12)	C17—O1—C19—C20	-80.99 (15)
C2—N2—C3—C4	81.94 (14)	Eu1—O1—C19—C20	119.23 (10)
Eu1—N2—C3—C4	-45.80 (13)	O1—C19—C20—C25	118.30 (15)
N2—C3—C4—N3	58.87 (16)	O1—C19—C20—C21	-64.19 (17)
C3—C4—N3—C28	76.93 (15)	C25—C20—C21—C22	0.3 (2)
C3—C4—N3—C5	-164.90 (12)	C19—C20—C21—C22	-177.27 (13)
C3—C4—N3—Eu1	-39.61 (14)	C20—C21—C22—C23	-0.9 (2)
C28—N3—C5—C6	-160.04 (12)	C21—C22—C23—C24	0.8 (2)
C4—N3—C5—C6	79.32 (14)	C21—C22—C23—B1	-179.10 (14)
Eu1—N3—C5—C6	-46.28 (13)	C22—C23—C24—C25	0.0 (2)
N3—C5—C6—N4	56.20 (16)	B1—C23—C24—C25	179.81 (14)
C5—C6—N4—C30	79.55 (15)	C21—C20—C25—C24	0.4 (2)
C5—C6—N4—C7	-161.68 (12)	C19—C20—C25—C24	177.88 (14)
C5—C6—N4—Eu1	-37.17 (14)	C23—C24—C25—C20	-0.5 (2)
C30—N4—C7—C8	-160.39 (13)	C24—C23—B1—O3	-16.2 (3)
C6—N4—C7—C8	80.10 (15)	C22—C23—B1—O3	163.65 (17)
Eu1—N4—C7—C8	-46.68 (15)	C24—C23—B1—O2	165.55 (15)
C9—N1—C8—C7	77.82 (15)	C22—C23—B1—O2	-14.6 (2)
C1—N1—C8—C7	-165.52 (12)	C2—N2—C26—C27	-168.44 (11)
Eu1—N1—C8—C7	-41.28 (14)	C3—N2—C26—C27	71.15 (14)
N4—C7—C8—N1	61.17 (17)	Eu1—N2—C26—C27	-45.78 (12)
C8—N1—C9—C10	-169.51 (12)	N2—C26—C27—O5	-161.15 (13)
C1—N1—C9—C10	72.57 (15)	N2—C26—C27—O4	20.55 (17)
Eu1—N1—C9—C10	-48.69 (13)	N2—C26—C27—Eu1	36.04 (9)
C18—N5—C10—C11	-3.5 (2)	O5—C27—O4—Eu1	-153.36 (12)
Eu1—N5—C10—C11	170.76 (11)	C26—C27—O4—Eu1	24.78 (16)
C18—N5—C10—C9	174.44 (12)	C4—N3—C28—C29	-166.75 (11)
Eu1—N5—C10—C9	-11.31 (16)	C5—N3—C28—C29	74.28 (13)
N1—C9—C10—N5	42.06 (18)	Eu1—N3—C28—C29	-44.94 (11)
N1—C9—C10—C11	-139.96 (14)	N3—C28—C29—O7	-158.74 (12)

N5—C10—C11—C12	-1.6 (2)	N3—C28—C29—O6	22.31 (17)
C9—C10—C11—C12	-179.47 (15)	N3—C28—C29—Eu1	34.69 (9)
C10—C11—C12—C13	3.7 (2)	O7—C29—O6—Eu1	-159.13 (11)
C11—C12—C13—C14	178.26 (15)	C28—C29—O6—Eu1	19.76 (16)
C11—C12—C13—C18	-0.8 (2)	C6—N4—C30—C31X	-174.6 (2)
C12—C13—C14—C15	-178.31 (14)	C7—N4—C30—C31X	65.9 (2)
C18—C13—C14—C15	0.8 (2)	Eu1—N4—C30—C31X	-51.8 (2)
C13—C14—C15—C16	3.2 (2)	C6—N4—C30—C31	-152.9 (2)
C14—C15—C16—C17	-2.1 (2)	C7—N4—C30—C31	87.6 (2)
C15—C16—C17—O1	174.48 (13)	Eu1—N4—C30—C31	-30.0 (2)
C15—C16—C17—C18	-3.0 (2)	N4—C30—C31—O9	-178.5 (3)
C10—N5—C18—C13	6.53 (19)	N4—C30—C31—O8	-3.0 (4)
Eu1—N5—C18—C13	-167.54 (10)	N4—C30—C31—Eu1	24.27 (17)
C10—N5—C18—C17	-171.30 (12)	O9—C31—O8—Eu1	-141.1 (3)
Eu1—N5—C18—C17	14.62 (16)	C30—C31—O8—Eu1	43.8 (3)
C12—C13—C18—N5	-4.4 (2)	Eu1—O8—C31X—O9X	-175.9 (3)
C14—C13—C18—N5	176.46 (13)	Eu1—O8—C31X—C30	-8.6 (5)
C12—C13—C18—C17	173.41 (13)	N4—C30—C31X—O9X	-147.8 (4)
C14—C13—C18—C17	-5.7 (2)	N4—C30—C31X—O8	44.0 (4)
O1—C17—C18—N5	6.99 (17)		

Hydrogen-bond geometry (Å, º)

D—H···*A*	*D*—H	H···*A*	*D*···*A*	*D*—H···*A*
C1—H1*B*···O5^{i}	0.99	2.44	3.3527 (18)	153
C4—H4*A*···O9^{ii}	0.99	2.35	3.317 (3)	164
C4—H4*A*···O9*X*^{ii}	0.99	2.28	3.244 (3)	165
C9—H9*B*···O11^{iii}	0.99	2.61	3.413 (2)	139
C11—H11···O2^{iv}	0.95	2.62	3.519 (2)	157
C19—H19*A*···O6	0.99	2.41	3.1619 (18)	132
O2—H2···O12^{v}	0.83 (1)	1.87 (1)	2.7009 (19)	178 (3)
O3—H3···O10	0.82 (1)	1.94 (2)	2.753 (2)	173 (3)
C26—H26*B*···O5^{i}	0.99	2.39	3.2838 (19)	149
C28—H28*B*···O4	0.99	2.64	3.1515 (18)	112
O10—H10*B*···O9*X*^{vi}	0.83 (1)	2.31 (2)	2.934 (4)	132 (2)
O10—H10*A*···O11^{vii}	0.81 (1)	2.19 (3)	2.769 (2)	129 (3)
O11—H11*A*···O10^{vii}	0.82 (1)	1.97 (2)	2.769 (2)	165 (3)
O11—H11*B*···O7	0.82 (1)	1.99 (1)	2.8081 (18)	171 (3)
O12—H12*A*···O13^{vii}	0.85 (1)	1.85 (2)	2.6891 (19)	172 (3)
O12—H12*B*···O7	0.85 (1)	1.93 (1)	2.7689 (19)	174 (3)
O13—H13*A*···O5	0.85 (1)	1.85 (1)	2.6973 (17)	176 (3)

O14—H14*A*···O15	0.82 (1)	1.89 (1)	2.701 (2)	172 (3)
O14—H14*B*···O10	0.83 (1)	1.95 (2)	2.765 (2)	168 (3)

Symmetry codes: (i) -*x*, -*y*+1, -*z*+1; (ii) -*x*+1/2, *y*-1/2, -*z*+1/2; (iii) -*x*+1/2, *y*+1/2, -*z*+1/2; (iv) *x*-1, *y*, *z*; (v) -*x*+3/2, *y*+1/2, -*z*+1/2; (vi) *x*+1/2, -*y*+3/2, *z*+1/2; (vii) -*x*+1, -*y*+1, -*z*+1.

Document origin: *publCIF* [Westrip, S. P. (2010). *J. Apply. Cryst.*, **43**, 920-925].

Computing details

Data collection: Bruker *APEX* 2; cell refinement: Bruker *SAINT* v8.38a; data reduction: Bruker *SAINT* v8.38a; program(s) used to solve structure: SHELXT 2014/5 (Sheldrick, 2014); program(s) used to refine structure: *SHELXL2018*/3 (Sheldrick, 2018); molecular graphics: Bruker *SHELXTL*; software used to prepare material for publication: Bruker *SHELXTL*.

Special details

Geometry. All esds (except the esd in the dihedral angle between two l.s. planes) are estimated using the full covariance matrix. The cell esds are taken into account individually in the estimation of esds in distances, angles and torsion angles; correlations between esds in cell parameters are only used when they are defined by crystal symmetry. An approximate (isotropic) treatment of cell esds is used for estimating esds involving l.s. planes.

References

1 D. Maffeo and J. A. G. A. G. Williams, Inorganica Chim. Acta, 2003, **355**, 127–136.

2 R. Radi, J. Biol. Chem., 2013, **288**, 26464–26472.

3 J. Tian, X. Yan, H. Yang and F. Tian, RSC Adv., 2015, **5**, 107012–107019.

4 A. Beeby, I. M. Clarkson, R. S. Dickins, S. Faulkner, D. Parker, L. Royle, A. S. de Sousa, J. A. G. Williams and M. Woods, J. Chem. Soc. Perkin Trans. 2, 1999, 493–504.

5 APEX 2 (2016) software for CCD diffractometers. Bruker AXS Inc., Madison, USA.

6 SAINT (2016) software for CCD diffractometers. Bruker AXS Inc., Madison, USA.

7 G.M. Sheldrick, Acta Cryst. 2015, **A71,** 3-8.

8 G.M. Sheldrick, Acta Cryst. 2015, **C71,** 3-8.

9 a) Spek, A. L. Acta Cryst. 2015, **C71**, 9-18; b) P.v.d. Sluis and A.L. Spek, Acta Cryst. 1990, **A46**, 194-201
